# Emergence of local and global synaptic organization on cortical dendrites

**DOI:** 10.1038/s41467-021-23557-3

**Published:** 2021-06-28

**Authors:** Jan H. Kirchner, Julijana Gjorgjieva

**Affiliations:** 1 Computation in Neural Circuits Group, Max Planck Institute for Brain Research, Frankfurt, Germany; 2 School of Life Sciences, Technical University of Munich, Freising, Germany

**Keywords:** Computational neuroscience, Synaptic plasticity

## Abstract

Synaptic inputs on cortical dendrites are organized with remarkable subcellular precision at the micron level. This organization emerges during early postnatal development through patterned spontaneous activity and manifests both locally where nearby synapses are significantly correlated, and globally with distance to the soma. We propose a biophysically motivated synaptic plasticity model to dissect the mechanistic origins of this organization during development and elucidate synaptic clustering of different stimulus features in the adult. Our model captures local clustering of orientation in ferret and receptive field overlap in mouse visual cortex based on the receptive field diameter and the cortical magnification of visual space. Including action potential back-propagation explains branch clustering heterogeneity in the ferret and produces a global retinotopy gradient from soma to dendrite in the mouse. Therefore, by combining activity-dependent synaptic competition and species-specific receptive fields, our framework explains different aspects of synaptic organization regarding stimulus features and spatial scales.

## Abstract (web summary)

Synaptic inputs on neuronal dendrites exhibit remarkable organization at different spatial scales, which emerges during the early postnatal development. Kirchner and Gjorgjieva propose a biophysically motivated computational model to explain the different types of organization in mouse and ferret.

## Introduction

Neurons in the developing brain become precisely connected before sensory organs mature. Spontaneous activity plays a major role in refining circuit connectivity to mature levels at the scale of single neurons and networks^1^. During visual system development, for example, synaptic connections are established by matching molecular gradients and axonal targeting^2^. Spatiotemporal correlations in spontaneous activity then instruct the development of various receptive field properties and visual feature maps, which are further fine-tuned by sensory activity^3^. In addition to refining developing networks at cellular precision, spontaneous activity can also establish fine-scale organization of individual synapses within the dendritic arborizations of single neurons^4,5^. One striking example of such fine-scale organization is functional synaptic clustering: synapses onto dendrites of pyramidal neurons that receive correlated input or encode a common sensory feature are spatially grouped. Synaptic clustering has been observed across brain regions, developmental ages, and diverse species from rodent to primate^4–12^, and has multiple functional benefits; it compartmentalizes the dendrites of single neurons, enables supralinear integration of inputs, shapes memory formation^13^, and can explain translation-invariance of complex cells in the visual cortex^14^. However, the mechanistic origins of synaptic clustering dependent on spontaneous activity during early postnatal development, and its relation to functional organization in the adult, remain elusive.

During development, recent experiments identified a molecular mechanism for the emergence of synaptic clustering based on the antagonistic interaction of brain-derived neurotrophic factor (BDNF) and its immature form, proBDNF. By binding to their corresponding receptors, BDNF and proBDNF can respectively promote synaptic potentiation and the survival of neurons, and synaptic depression and the apoptosis of axons and neurons^15^. While proBDNF is more prevalent than BDNF during early development^16^, the protease matrix metallopeptidase 9 (MMP9) controls the relative amounts of proBDNF and BDNF at a given synapse in an activity-dependent manner, and therefore regulates the plasticity of that synapse. This sets up a promising mechanistic implementation for the developmental formation of synaptic clusters based on neurotrophin interactions, yet the key computational properties that lead to the activity-dependent cooperation and competition of multiple synapses innervating a dendritic branch are unknown.

In the adult, several in vivo studies have reported clustering of different stimulus features in different species (but see refs. ^17–20^). For example, dendritic branches in the ferret visual cortex exhibit local clustering of orientation selectivity but do not exhibit global organization of inputs according to spatial location and receptive field properties^7,9^. In contrast, synaptic inputs in the mouse visual cortex do not cluster locally by orientation, but only by receptive field overlap, and exhibit a global retinotopic organization along the proximal-distal axis^8^. We presently do not understand the factors that underlie these scale- and species-specific differences and how they emerge during development.

Here we propose a computational framework to reconcile experimental findings about the fine-scale and global synaptic organization observed in the adult, and to make predictions about the key factors driving the emergence of this organization during development. We built a biophysically inspired model of synaptic plasticity based on the molecular mechanism of interacting neurotrophins required for synaptic clustering during development^6,10^. We identified two important ingredients necessary to generate clustering with this model: timing-dependent cooperation and distance-dependent competition. Generalizing this neurotrophin model to an analytically tractable framework with these two characteristics enables us to study the emergence of synaptic organization independent of the specific mechanistic implementation. When stimulated with spontaneous retinal waves in a realistic scenario of visual system development, the model generates clustering by orientation in the adult ferret visual cortex and clustering by receptive field overlap in the adult mouse visual cortex. Two key parameters determine the type of clustering: the diameter of receptive fields and the spread of receptive field centers in visual space, which depends on the cortical magnification factor of visual space. By introducing a backpropagating action potential to a reconstructed dendritic tree, the same model generates global organization across the entire tree. Therefore, a single computational framework motivated by molecular interactions in development can explain how circuits wire with the remarkable subcellular precision observed in adulthood, integrating many different facets of organization at the local and global scale.

## Results

### Distance- and timing-dependent synaptic plasticity in an activity-dependent neurotrophin model

**Fig. 1:**
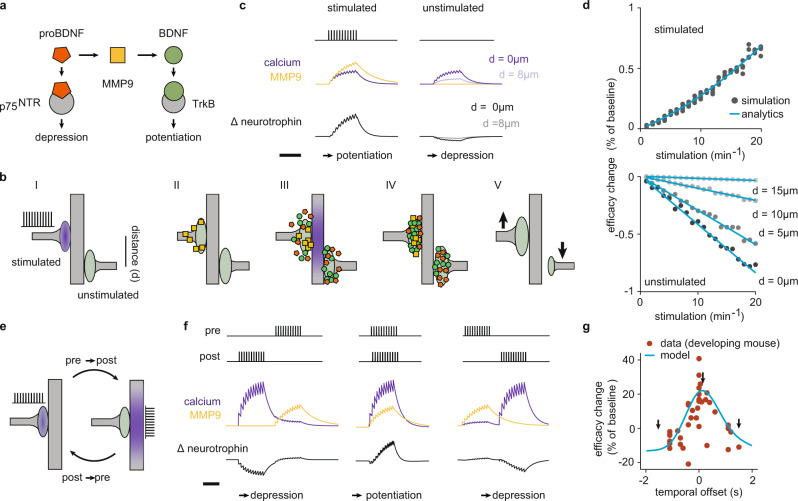
Distance- and timing-dependent synaptic competition in the neurotrophin model. **a** Model schematic: interactions between neurotrophins (BDNF and proBDNF), neurotrophin receptors (TrkB and P75^NTR^), and cleaving protease (MMP9). **b** Outcome of synaptic stimulation where two synapses separated by a distance *d* innervate the same dendrite: I. The left synapse is stimulated with a burst of action potentials. II. Presynaptic activation causes the local release of MMP9 (yellow). III. Signal transduction into the postsynapse results in the spatially extended influx of calcium (purple shading). Calcium triggers the exocytosis of proBDNF (orange) and BDNF (green) into extracellular space. IV. MMP9 differentially cleaves proBDNF into BDNF at the stimulated synapse. V. Repeating this pattern of stimulation potentiates the stimulated synapse and depresses the unstimulated synapse (arrows). Symbols as in **a**. **c** Variables (all unitless, see Methods) in **a** upon stimulation of one synapse only; the unstimulated synapse is distance *d* away, as in **b**. $\varDelta$ neurotrophin is the difference between BDNF and proBDNF. Scale bar is 1 s. **d** Percentage change in synaptic efficacy (of baseline) of the stimulated synapse (top) and the unstimulated synapse (bottom) as a function of input rate (in bursts per minute, after 1 min of continuous stimulation) and distance *d*. The analytical solution follows from a linearized version of the model (see Methods). **e** A burst-timing-dependent plasticity induction protocol where the temporal offset between pre- and postsynaptic bursts is varied^27^. A single synapse innervates the dendrite. Purple shading indicates either pre- (left) or postsynaptic (right) activation. **f** Accumulator and neurotrophin variables under the burst pairing protocol with temporal offsets −1.5 s (left), 0.05 s (middle), and 1.5 s (right). Scale bar is 0.5 s. We assumed that the calcium from direct postsynaptic stimulation is stronger than that released by stimulation of a synapse (compare to **c** and see Methods). **g** Percentage change in synaptic efficacy (of baseline) as a function of temporal offset (data repesent change in EPSP size from developing mouse LGN reproduced from ref. ^27^). The arrows mark the temporal offsets displayed in **f**.

To identify the driving factors for fine-scale dendritic organization of synaptic inputs, we formulated a computational model based on a local molecular mechanism implicated in the emergence of clustering during development. The mechanism implements an activity-dependent interaction between two signaling molecules: proBDNF and BDNF, and the conversion factor between them, MMP9 (Fig. 1a and Methods). Upon activation, a synapse in our model evokes the postsynaptic release of proBDNF and BDNF through the opening of voltage-gated calcium channels and the subsequent influx of calcium^10^, which spreads postsynaptically in the developing brain^21^. Through the lateral spread of calcium, the activated synapse can exert a direct effect on a different nearby synapse by triggering neurotrophin release independent of presynaptic stimulation of that synapse. MMP9 release is also coupled to neural activity^22^, but instead of spreading postsynaptically, it is co-localized with glutamatergic receptors in excitatory synapses^23^. Therefore, we modeled MMP9 and calcium as synapse-specific and shared accumulators of neural activity, respectively (see Methods).

Analyzing the molecular interactions in the neurotrophin model revealed two prominent properties for sorting dendritic synaptic inputs into local clusters: timing-dependent cooperation and distance-dependent competition. Distance-dependent competition arises when considering the plasticity of two nearby synaptic inputs as a function of the distance between them and the stimulation frequency upon stimulating one of them (Fig. 1b–d). Driving one synapse with bursts, the units of information transmission during development^6,24^, shifts the balance between proBDNF and BDNF^25^ in favor of BDNF due to the activity-dependent release of MMP9 (Fig. 1b, c). Thus, the stimulated synapse potentiates. In contrast, the unstimulated synapse depresses by an amount depending on the distance between synapses, as it remains dominated by proBDNF due to the absence of MMP9 (Fig. 1d and Methods). Plasticity of an unstimulated synapse following the stimulation of another synapse is called heterosynaptic and can stabilize the positive feedback of Hebbian plasticity^26^.

In addition to this distance-dependent heterosynaptic depression, the neurotrophin model exhibits timing-dependent cooperation by potentiating nearby coactive synapses. This can be best seen when implementing a classical plasticity induction paradigm where we quantified synaptic strength as a function of the timing between pre- and postsynaptic events (Fig. 1e). Using bursts as the natural activity patterns in development^6,24^, we found that pre- and postsynaptic bursts with temporal offsets below 1 s yield synaptic potentiation due to the relative dominance of BDNF over proBDNF-induced signaling, while longer offsets lead to depression (Fig. 1f, g). This type of plasticity in the neurotrophin model can be matched to a timing-dependent Hebbian learning rule described in the developing visual system, burst-timing-dependent plasticity (BTDP)^27^. According to this rule, synaptic change is sensitive to the overlap between pre- and postsynaptic bursts on the timescale of several hundred milliseconds. Remarkably, the dependence of the neurotrophin model on burst-timing as in the BTDP rule is robust to perturbations in most parameters of the model except for the proBDNF/BDNF ratio, which can shift the BTDP curve into depression and eliminate synaptic competition (Supplementary Fig. 1).

In summary, the proposed neurotrophin model provides a mechanistic implementation for (1) the distance-dependent competition between differentially stimulated synapses and (2) the timing-dependent potentiation when pre- and postsynaptic activity overlap over developmentally relevant timescales of several hundred milliseconds. Identifying these two ingredients provides a key step towards a general framework for establishing synaptic organization in development^6,10^ and reconciling different aspects of synaptic organization observed in the adult^7–9^.

### A generalized neurotrophin-inspired model captures out-of-sync-lose-your-link plasticity

To establish a framework for synaptic organization, we generalized the neurotrophin model to a local dendritic learning rule independent from a specific biophysical implementation. The generalized model derives directly from neurotrophin interactions, and hence retains the two key properties of timing- and distance-dependent plasticity (Supplementary Fig. 2). Synaptic efficacy change depends on the accumulated presynaptic (pre) and postsynaptic (post) activity and a constant heterosynaptic offset related to the initial ratio of proBDNF to BDNF in the absence of extracellular conversion through MMP9 (see Methods),

1 $${\mathrm{change}}\,{\mathrm{in}}\,{\mathrm{synaptic}}\,{\mathrm{efficacy}}={\rm{post}}\times ({\rm{pre}}-{\rm{offset}})$$

This generalized neurotrophin-inspired model has the advantage that it can be analyzed mathematically, can be flexibly implemented to apply to other signaling molecules, and hence, a broader cast of synaptic organization scenarios. This is especially important since the role of neurotrophins in the emergence of clustering of different stimulus features, as well as their interaction with plasticity-related proteins involved in tag-and-capture and synaptic crosstalk^28–32^ in adulthood, is unknown.

To determine how distance- and timing-dependent plasticity might drive the emergence of synaptic organization with respect to different stimulus features, we next investigated the consequences of these two model characteristics on the organization of multiple randomly distributed synaptic inputs on a linear dendritic branch. Under the assumption that synaptic efficacy changes on a much slower timescale than neural activity, the average change in synaptic efficacy can be expressed as a function of the synaptic input correlation (Eq. 11 in Methods). Therefore, the timing-dependent plasticity of synaptic inputs represented by the BTDP rule in our model translates into local plasticity that depends on input correlations^33^. To investigate distance-dependent competition of multiple randomly distributed synaptic inputs on a dendritic branch, we varied the synaptic density. Locally, low synaptic density implies that pairs of synaptic inputs are on average far away from each other, while high synaptic density implies that pairs of synaptic inputs are on average near each other. Hence, local density relates to synaptic distance.

**Fig. 2:**
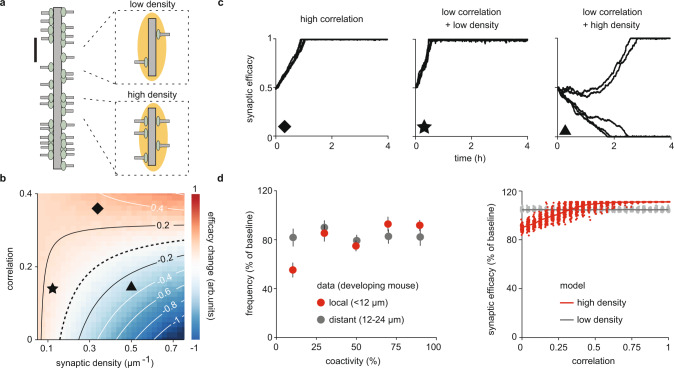
Relating synaptic efficacy change to synaptic density and input correlation. **a** Schematic with randomly distributed synapses along a linear dendritic branch. All synapses receive Poisson input trains with identical pairwise correlations. Scale bar is 20 μm. **b** Average instantaneous change in synaptic efficacy as a function of input correlation and density. Contour lines are determined by analytics, while colors by simulation, averaging over time and synapses (negative synaptic efficacy change in blue, positive synaptic efficacy change in red). **c** Evolution of synaptic efficacy for five different synaptic inputs at various combinations of density and correlation as in **b** (diamond, star, and triangle at densities 0.312, 0.125, and 0.5 μm^−1^ and correlations 0.15, 0.35, and 0.15, respectively). For low correlation and high density, individual efficacy trajectories diverge due to random fluctuations in the inputs. As some synapses depress, the effective density decreases (the triangle moves to the left from a depression to a potentiation region as shown in **b**). **d** Left: Percentage change in synaptic transmission frequency after plasticity in the developing visual cortex relative to baseline as a function of coactivity for local (red) vs. distant (gray) synapses. Data from developing mouse visual cortex reproduced from ref. ^6^ and presented as mean values ± SEM (*n* = 11 cells, pooled data of 352 synapses). Right: Percentage change in synaptic efficacy (of baseline) in our model as a function of correlation for synapses at high density (0.5 μm^−1^, a synapse every 2 μm, red) and low density (0.05 μm^−1^, a synapse every 20 μm, gray). Dots represent individual synapses.

We derived the average change in synaptic efficacy as a function of two parameters: the input correlation and the synaptic density on the dendritic branch (Fig. 2a, b and Supplementary Fig. 2). We identified three regimes: (i) at any synaptic density, if the synaptic input correlation is higher than a critical amount (Methods) then synapses stabilize (Fig. 2c, diamond), (ii) at low synaptic density, if the input correlation is lower than the same critical amount then synapses also stabilize (Fig. 2c, star), and (iii) at high synaptic density, if the input correlation is lower than the critical amount then synapses compete (Fig. 2c, triangle). Note that in regime (iii), synapses compete until a random subset is silenced, which reduces the effective density of the remaining synapses and eventually stabilizes them (Fig. 2b). The proposed generalization of the neurotrophin model thus implements Hebbian correlation-based plasticity at the single synapse^34^ where synaptic efficacy is modulated by the local density of synaptic inputs on the dendrite, just like the implementation with interacting neurotrophins (Supplementary Fig. 2).

The generalized neurotrophin-inspired model for clustering enabled us to dissect how specific neurotrophin interactions influence the functional dependence of synaptic efficacy on input correlation and synaptic density. In particular, it is well established that BDNF can increase, while proBDNF can decrease the synaptic density on developing dendrites^15^. Applying those same perturbations to our model produces similar neurotrophin-induced changes in synaptic density, since the ratio of BDNF to proBDNF determines the synaptic density at which potentiation and depression are balanced through the heterosynaptic offset (Supplementary Fig. 3).

Additionally, the generalized model remains relevant for the emergence of synaptic organization in development. Key properties that are accounted for are the slow timescales of the pre- and postsynaptic accumulators that respectively correspond to the time constants of MMP9 and postsynaptic calcium and give rise to the developmental BTDP plasticity rule (Supplementary Fig. 2). Additionally, the model implements the lateral postsynaptic spread of calcium, consistent with developmentally prevalent shaft synapses which can interact over long distances more easily than adult spine synapses^35^.

As a result, the synaptic competition in our model (Fig. 2b, c) resembles a local out-of-sync-lose-your-link plasticity rule underlying the emergence of clustering in the developing visual cortex and hippocampus^6^. According to this rule, synaptic inputs on developing pyramidal dendrites either increase or decrease the success rate of synaptic transmission depending on their synchrony with their nearby, but not distant, neighbors (Fig. 2d, left). To directly compare our model to this experimental data, we visualized the synaptic efficacy as a measure of synaptic transmission success at two different densities (Fig. 2b): a high density of 0.5 μm^−1^ (a synapse every 2 μm), which corresponds to local synapses in the data (<12 μm), and a low density of 0.05 μm^−1^ (a synapse every 20 μm), which corresponds to distant synapses in the data (12–24 μm). Our model generates a similar out-of-sync-lose-your-link rule where local synaptic inputs (high density) are depressed if weakly correlated and potentiated if strongly correlated. In contrast, distant synaptic inputs (low density) are stabilized independent of correlation (Fig. 2d, right). A similar out-of-sync-lose-your-link structural plasticity, without the local postsynaptic component, has also been studied previously to solve more abstract classification tasks^36–38^.

Taken together, our generalized model based on neurotrophin interactions with properties of timing- and distance-dependent competition provides a general framework to study the emergence of synaptic organization on dendritic branches innervated by multiple synaptic inputs. The model relates synaptic efficacy change to synaptic input correlation and local density and can capture the depression of weakly correlated neighboring inputs embodying out-of-sync-lose-your-link plasticity, implicated in the emergence of functional synaptic clustering during development.

### Retinal wave input and synaptic turnover drive synaptic orientation clustering in a model of the ferret visual cortex

**Fig. 3:**
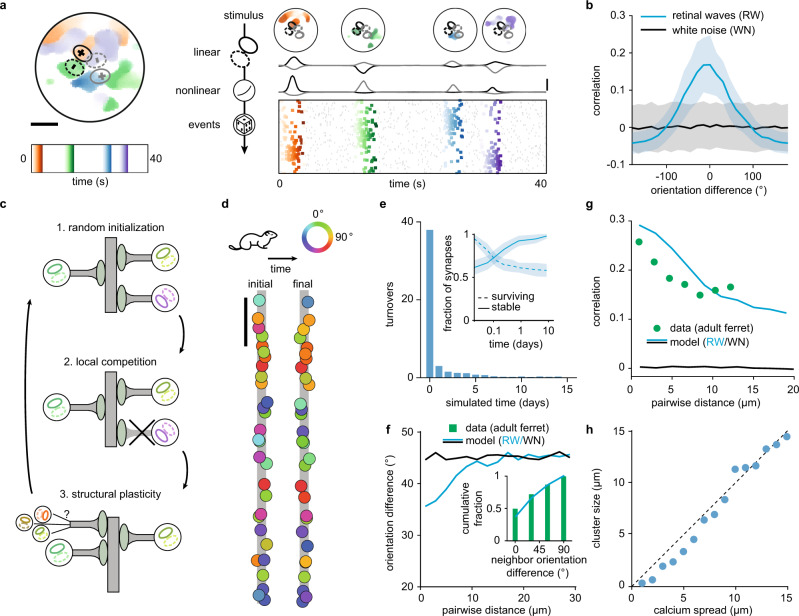
Retinal wave input and synaptic turnover produce orientation clustering on a dendritic branch in a model of the ferret visual cortex. **a** (Left) Illustration of 40 s of retinal wave input. The color gradients indicate different time points. Scale bar is 30° in visual space. Superimposed are two schematics of Gabor receptive field filters with positive (full line) and negative (dashed line) components. (Right, from top) Four spontaneous retinal waves as stimuli, linearly filtered responses, and nonlinear outputs for the two oriented Gabors on the left. Scale bar is 1 Hz. Rows in raster plot correspond to firing events from different receptive fields, sorted from top to bottom by orientation. Gray events represent background noise. **b** Mean correlation as a function of the difference in orientation of receptive fields stimulated with retinal waves (RW) or white noise (WN) input. Shaded area indicates standard deviation. **c** Local correlation- and distance-dependent competition, together with structural plasticity, drive the formation of synaptic clusters. **d** Example of the emergence of orientation clustering on a linear dendrite using ferret cortex receptive field diameter and center distribution with retinal waves over 2 simulated weeks. Color of synapses indicates the orientation preference of the associated receptive field. Scale bar is 20 μm. **e** Number of turnovers as a function of simulated days. Inset: Semi-log plot of the mean survival fraction (synapses present at the beginning of simulation) and the stable fraction (synapses present at the end of the simulation) as a function of simulated days. Shaded area indicates standard deviation. **f** Orientation difference between pairs of synapses as a function of distance in the model with retinal waves or white noise. Inset: Cumulative fraction of orientation difference between an individual synapse and its nearby neighbors (less than 3 μm away) in the model and experiments (data from adult ferret visual cortex reproduced from ref. ^7^). Difference computed after taking the orientation modulo 180° (see Methods). **g** Correlation between pairs of synaptic inputs in the model driven by retinal waves or white noise, and correlation between calcium signals of spontaneous synaptic activity (data from adult ferret visual cortex reproduced from ref. ^9^) as a function of distance. **h** Cluster size computed as the standard deviation of the best Gaussian fit to the pairwise correlation vs. distance (see Methods and **g**), as a function of the postsynaptic calcium spread. Dashed line indicates the identity line.

We next tested the potential of the above-developed model inspired by neurotrophin interactions to organize synaptic inputs in the developing visual cortex driven by spontaneous activity propagated from the sensory periphery^39,40^. We simulated retinal waves^41^ and converted them to cortical synaptic input via a two-stage linear-nonlinear (LN) model (Fig. 3a and Methods). For the linear filter we used a spatially oriented Gabor with positive and negative elongated subregions based on receptive field measurements of individual spines on pyramidal neurons in the adult mouse visual cortex^8^. Each Gabor receptive field is characterized by three parameters: center, described in polar coordinates, diameter, and orientation, with a value between 0° and 360° (Fig. 3a). We inferred the receptive field diameter and center distribution for the visual cortex of two species, mouse and ferret (see Methods). An exponential nonlinearity converts the linearly filtered retinal waves into instantaneous firing rates from which activity is generated via a Poisson process (see Methods). Thus, in the LN model, a synapse probabilistically receives bursts of action potentials when the Gabor filter is stimulated by a retinal wave traveling in the direction that matches the filter orientation (Fig. 3a). Since activation of a synapse depends on the appropriate stimulation of its associated receptive field, synapses with nearby receptive field centers in visual space and a small difference in orientation experience correlated input (Fig. 3b). White noise stimulation does not produce correlations for any orientation difference due to the lack of spatiotemporal structure to consistently activate nearby receptive fields (Fig. 3b).

**Table 1:** Parameters of the proposed model along with nominal values used for simulations, unless stated otherwise.

Parameter	Variable	Value
Synaptic efficacy time constant	${\tau }_{W}$	6 s
proBDNF and BDNF time constants^31^	${\tau }_{P},{\tau }_{B}$	5 ms
Postsynaptic calcium time constant^21^	${\tau }_{Y}$,${\tau }_{u}$	300 ms
MMP9 time constant^83^	${\tau }_{M}$,${\tau }_{v}$	600 ms
Constitutive percent of BDNF of amount of total neurotrophins released^25^	$\eta$	45%
MMP9 efficiency constant^22^	$\phi$	$\frac{3}{50}$ ms^−1^
Heterosynaptic offset*	$\rho$	$\frac{2\eta -1}{2(1-\eta )}$
Synaptic efficacy time constant in the generalized neurotrophin-inspired model*	${\tau }_{w}$	${\tau }_{W}\frac{1}{2(1-\eta )}$
Standard deviation of calcium spread^21^	${\sigma }_{c}$	6 μm
Density of synapses^85^	$\nu$	0.2 μm^‒1^
Spread of receptive field centers for ferret and mouse^8,9^, in visual space	${\sigma }_{p}$	5.3°, 26°
Diameter of Gabor receptive field mouse^8^, ferret^9^, and macaque^12^		20°, 13.4°, 2°
LN model parameters^6,9^	$a,b$	0.2 Hz, 9.4
Turnover threshold below which a synapse is replaced	${W}_{{\rm{thr}}}$	0.02
Threshold for bAP generation^48^	${B}_{{\rm{thr}}}$	25
Unattenuated amplitude of bAP^47,48^	${B}_{{\rm{amp}}}$	5

Citations indicate a free parameter fitted to experimental data.

*Indicates a parameter that is derived from the other parameters.

Using a receptive field diameter and a center distribution from the ferret visual cortex^9^, we placed synaptic inputs with randomly oriented receptive fields on a non-branching, linear dendrite—as a model of small, approximately linear portions of real dendritic trees —and stimulated them with retinal waves^41^ filtered through an LN model. Neighboring synapses with mismatched orientations receive uncorrelated input as they are rarely activated by the same retinal wave and consequently depress. To prevent their irrevocable elimination, we modeled an activity-dependent mechanism of structural plasticity, which preserves the total number of synaptic inputs on a dendritic branch^42,43^ (Fig. 3c). Upon the removal of a synapse, a new synapse is placed at a random position on the dendritic branch with a randomly oriented receptive field and center in visual space sampled from the experimentally measured distribution in the ferret visual cortex^9^ (see Methods).

Throughout the simulation, synapses compete and are either stabilized or eliminated and turned over until nearby synapses share a similarly oriented receptive field (Fig. 3d). The number of turnovers per day decreases rapidly, with all remaining turnovers due to a small fraction of synapses (Fig. 3e). Despite substantial turnover during the first three days of the simulation, ~60% of synapses present at the beginning of the simulation do not experience any turnover and form a scaffold around which the remaining synapses stabilize (Fig. 3e, inset). Synapses that do not experience any turnover throughout the entire simulation tend to have a smaller-than-chance orientation difference from each other.

We found that all nearby synapses in the stable state share similarly oriented receptive fields, a type of clustering that we call orientation clustering (Fig. 3d right, f). Orientation clustering has been found in layer 2/3 of the adult ferret visual cortex in vivo^7,9^. This orientation clustering further generates functional synaptic clustering in our simulations, where correlations between pairs of synapses trained with retinal wave input decay with distance at the same rate as during spontaneous activity in layer 2/3 of the adult ferret visual cortex (Fig. 3g). When using this relationship to characterize the size of the formed clusters, we found that cluster size strongly depends on the spatial spread constant of postsynaptic calcium (Fig. 3h and Supplementary Fig. 4). Therefore, we suggest that the different sizes of orientation clusters found in different species^9,12^, variability across different cells of the same animal^9^, as well as potential differences in development vs. adulthood, could be the result of different amounts of postsynaptic calcium spread. Orientation clustering also emerges when we implement the full complement of neurotrophin interactions in our model (Supplementary Fig. 2). While we do not know whether neurotrophins directly drive the orientation clustering observed in the adult brain, our results show that slow developmental timescales of plasticity and input correlations together with timing- and distance-dependent synaptic competition are sufficient.

Orientation and functional clustering do not emerge with white noise stimulation (Fig. 3f), nor with a spike-timing-based plasticity (STDP) rule that induces synaptic change based on precise spike timing (Supplementary Fig. 5), due to the mismatch of timescales between the input patterns and the induction of plasticity^24,27,33^. Including a dendritic nonlinearity preserves clustering and decreases the average nearest neighbor distance (Supplementary Fig. 6). Similarly, when requiring the synchronous activation of multiple neighboring synapses for the induction of plasticity^26,34^, clustering persists when the timing and spacing thresholds for such cooperative plasticity are consistently satisfied by the developmental slow timescales of activity and low synaptic density (Supplementary Fig. 6).

Otherwise, clustering is remarkably stable against perturbations of most model parameters (Supplementary Fig. 1). Clustering is, however, sensitive to changes in the heterosynaptic offset, which can shift plasticity into an exclusively potentiating or depressing regime (Supplementary Fig. 1). Since our model is based on neurotrophin interactions, we can interpret perturbations to the heterosynaptic offset as perturbations in the balance between BDNF and proBDNF signaling (Supplementary Fig. 3). For instance, exogenous application of either proBDNF or BDNF can abolish clustering in vitro within minutes^10^. Our model also makes predictions for how additional perturbations to the conversion between the two neurotrophins, or the receptors to which they bind, might affect synaptic efficacy and clustering (Supplementary Fig. 3).

In summary, with a synaptic receptive field diameter and a center distribution from the ferret visual cortex, our model generates local clusters of similarly oriented and, therefore, functionally correlated synaptic inputs on a dendritic branch based on correlated input from retinal waves. Therefore, a neurotrophin-inspired model for clustering supports the emergence of local synaptic organization in the ferret cortex.

### Clustering of different stimulus features in mouse and ferret

In contrast to the ferret, nearby synapses on pyramidal neuron dendrites in the mature mouse visual cortex do not share a preference for the same orientation^8,19,20,44^. However, nearby synapses in the mouse visual cortex still exhibit correlated activity during development^6^, and hence are functionally clustered. We next investigated whether our modeling framework can also generate the functional clustering observed in the mouse.

**Fig. 4:**
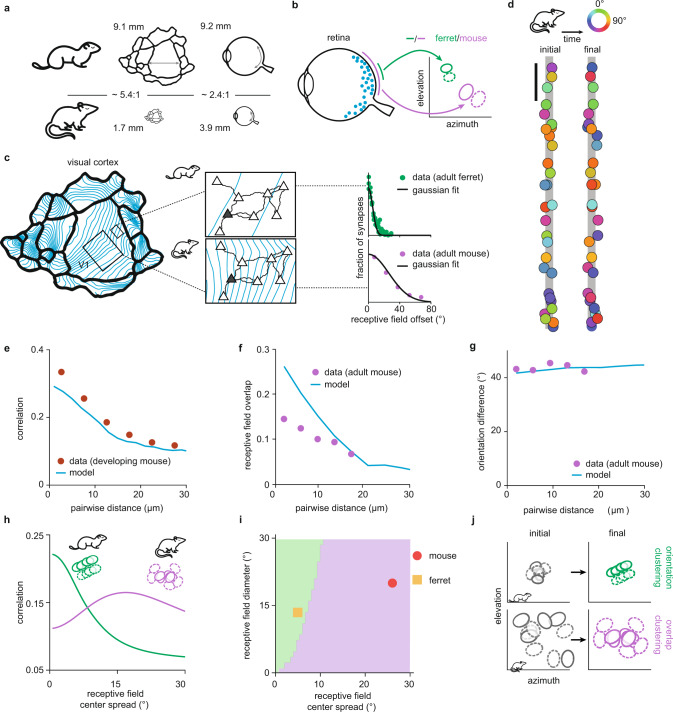
Overlap clustering, but not orientation clustering, emerges on a dendritic branch in a model of the mouse visual cortex. **a**–**c** Schematic of the anatomical argument for two qualitatively different types of clustering in mouse and ferret. Note that we depict ferret cortex as a scaled-version of mouse cortex. **a** Comparison of cortex and retina size in mouse and ferret (measures from ref. ^45^). **b** Schematic linking retina size (left) to receptive field diameter in the cortex (right). **c** Schematic linking cortex size to receptive field center spread. 1 mm of visual cortex spans a larger region of the total visual space (left, blue lines represent iso-contours at ~7° ^46^) in the mouse than in the ferret. Since the dendritic trees of pyramidal neurons (triangles) in the two species are comparable in size, a target neuron (gray) pools input from a smaller region of visual space in ferret (top middle) than in mouse (bottom middle). The synaptic receptive fields on the dendritic tree of the target neuron are distributed in a small (ferret, top right) or large (mouse, bottom right) region in visual space. The receptive field spread is quantified by the standard deviation of the distribution of their centers (5.3° for ferret and 26° for mouse, data reproduced from refs. ^8,9^). **d** Example demonstrating lack of orientation clustering on a linear dendrite using mouse cortex receptive field spread and diameter with retinal waves over 2 simulated weeks. Scale bar is 20 μm. **e** Correlation between pairs of synaptic inputs in the model driven by retinal waves, and correlation between calcium signals of spontaneous synaptic activity (data from developing mouse visual cortex reproduced from ref. ^6^) as a function of distance. **f** Receptive field overlap for pairs of synaptic inputs as a function of distance in the model and experiments (data from adult mouse visual cortex reproduced from ref. ^8^). **g** Orientation difference between pairs of synapses as a function of distance in the model and experiments (data from adult mouse visual cortex reproduced from ref. ^8^). **h** Average correlation between nearby synapses as a function of receptive field center spread for simulated synaptic receptive fields that are either orientation clustered (green) or overlap clustered (purple). **i** Varying receptive field center spread and diameter in our model dichotomizes the area in which orientation clustering (green) or overlap clustering (purple) produces higher average correlation (see Methods). **j** Schematic showing how orientation (top) and overlap (bottom) clustering emerge from a combination of receptive field diameter and center spread.

One striking difference between mouse and ferret is the size of their retina and visual cortex, with each being about two vs. five times smaller in the mouse than in the ferret, respectively^45^ (Fig. 4a). Consequently, the average receptive field diameter in the mouse visual cortex is about twice as large, and the cortical magnification of visual space about five times smaller than in the ferret^9^ (Fig. 4b, c). Consistent with these anatomical differences, a pyramidal neuron in the mouse visual cortex receives inputs from a considerably larger region of visual space than in the ferret^9^ (Fig. 4c, middle). The difference in the sampled region of visual space can be captured by the relative broadness of the distribution of receptive field centers characterized experimentally in the two species^8,9^, a parameter that we call the receptive field center spread (Fig. 4c, right). We implemented our neurotrophin-inspired model for clustering using a larger receptive field diameter and center spread as measured in the mouse visual cortex^8^ to determine the synaptic organization driven by retinal waves (Fig. 4d). We observed functional synaptic clustering during spontaneous activity, as measured during development^6^ (Fig. 4e). Interestingly, this functional organization is the result of synaptic clustering for receptive field overlap, rather than orientation (Fig. 4d–g). Such clustering of synapses with overlapping receptive fields is consistent with recent measurements in the adult mouse visual cortex^8^. We refer to this type of clustering as overlap clustering to contrast it with the orientation clustering observed in the ferret. Adding irregularities to the Gabor synaptic filters further improves the match between model and data (Supplementary Fig. 7).

To understand the principal factors that determine the type of clustering, we computed the correlations of synaptic activity resulting from the overlap of simulated synaptic receptive fields—that are either orientation clustered or overlap clustered—upon perturbing the spread of their centers on the dendritic branch (Fig. 4h). The orientation clustered receptive fields produce the highest correlations when the receptive field center spread is small (~5°, as in the ferret). With increasing spread, the overlap between the positive and negative components of the Gabor filter increases, which decreases correlations (Fig. 4h; green). Conversely, the receptive fields clustered by overlap produce the highest correlations when receptive field center spread is larger (~15–20°, as in the mouse) (Fig. 4h; purple).

Next, we determined the prevalence of orientation vs. overlap clustering by simultaneously varying the two anatomical parameters, the center spread, and the diameter of Gabor receptive fields in the visual cortex, and without modeling other differences in morphology, dendritic arborization, or synaptic density (Fig. 4i). Interestingly, for a very small receptive field diameter, only small receptive field center spread yields orientation clustering in our framework. Consistent with this, in the macaque visual cortex, which has a small receptive field diameter and receptive field center spread, orientation clustering has recently been reported^12^. Therefore, our model explains the emergence of synaptic clustering with respect to different stimulus features in the mouse and ferret. Without modeling other differences in the visual systems, we find that clustering emerges from maximizing the amount of correlation as a function of the geometric arrangement of synaptic receptive fields in the different species (Fig. 4j).

### Backpropagating action potentials establish global orientation clustering on ferret dendrites

**Fig. 5:**
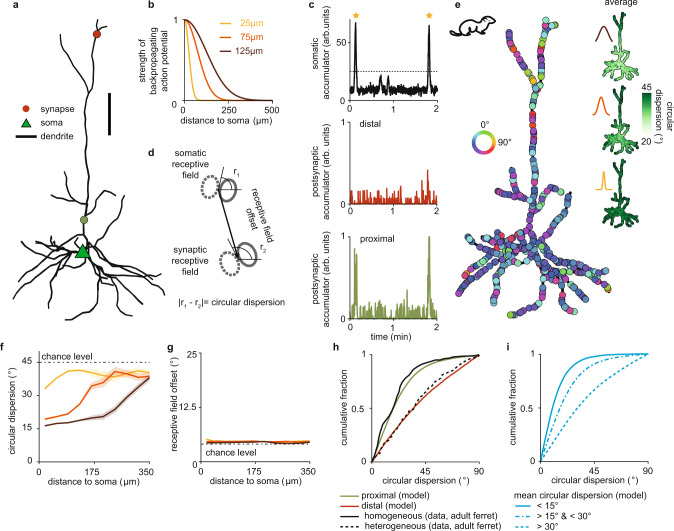
A backpropagating somatic signal drives heterogeneously and homogeneously clustered dendritic branches in a model of the ferret visual cortex. **a** Illustration of a reconstructed pyramidal cell from layer 2/3 (Allen Cell Type database, ID 502269786). Triangle indicates soma. Circles indicate synaptic sites. Scale bar is 50 μm. **b** Strength of backpropagating action potentials (bAPs) for different attenuation factors as a function of path distance from the soma. **c** Top: Sample trace of somatic activation under retinal wave stimulation after five simulated days. Asterisks indicate the initiation of multiple global somatic signals in the form of a burst of bAPs after threshold crossing (dashed line). Middle, Bottom: Postsynaptic calcium traces of a distal (middle) and a proximal (bottom) synapse (indicated in **a**). **d** Schematic to illustrate circular dispersion and receptive field offset (see Methods). **e** Emergence of global organization of orientation preference on the reconstructed pyramidal cell using ferret cortex receptive field spread and diameter. Color of synapses indicates the orientation preference of the associated receptive field. Inset shows the circular dispersion averaged over 62 simulations for the three different attenuation factors in (**b**). **f**, **g** Circular dispersion (**f**) and receptive field offset (**g**) between synapse and soma for the three different bAP attenuation factors in (**b**) and as a function of path distance from the soma. Shaded areas indicate 95% confidence interval around the mean. **h** Cumulative fraction of circular dispersion for one bAP attenuation factor (75 μm) in the model, and different types of clustered branches in experiments (data from adult ferret visual cortex reproduced from ref. ^7^). **i** Same as **h** for branches with low (<15°), intermediate (15°–30°), and high (>30°) mean circular dispersion in the model.

We next asked whether the local organization of synaptic inputs achieved by our model also supports the emergence of global organization of synaptic inputs on the dendritic tree. To probe interactions between the soma and synapses on different dendritic branches in a biologically realistic framework beyond the linear dendrites considered so far, we implemented our plasticity model on a morphologically realistic layer 2/3 pyramidal neuron (Fig. 5a). We modeled a somatic signal that affects the dendrite in the form of a backpropagating action potential (bAP) whenever the linearly summed input over all synapses exceeds a fixed threshold. The bAP results in a calcium influx into the proximal and distal dendritic branches that attenuates with distance from the soma (Fig. 5b, c)^47^. As a result, the calcium signal at proximal synapses in our model neuron is dominated by somatic activity, while distal synapses are almost independent of the soma (Fig. 5c) as found experimentally^48^.

Since bAPs are known to induce the release of neurotrophins^49^, we investigated how an attenuating bAP affects the correlation- and distance-dependent competition when implementing the full complement of neurotrophin interactions. We found that a bAP naturally extends both mechanisms over larger spatial scales, so that synapses near the soma experience stronger depression than those farther away from the soma when they are activated asynchronously to the soma, and stronger potentiation when activated in synchrony with the soma (Supplementary Fig. 8). Therefore, the addition of a bAP should not affect local synaptic organization on branches far away from the soma, but reinforce it near the soma.

Using a small receptive field diameter and center spread for the ferret visual cortex, our model generates local orientation clusters along the entire dendritic tree just like on the linear dendrite (Fig. 5d, e and Supplementary Fig. 9). By including bAPs, global organization on the entire dendritic tree also emerges. Synaptic inputs on proximal dendritic branches acquire orientation preferences similar to the soma due to the weak decay of bAP signaling, while inputs on distal dendrites have orientation preferences that are independent of the soma due to the strong bAP attenuation (Fig. 5e). Therefore, including bAPs homogenizes proximal dendritic branches to the orientation preference of the soma while leaving distal synapses unconstrained (Fig. 5e, inset), and hence variable across individual simulations (Supplementary Fig. 9). We characterized the degree of branch heterogeneity by computing the circular dispersion, i.e., the difference between the orientation preference of individual synapses and the soma (Fig. 5d and Methods). We found that bAP attenuation controls the extent of cluster homogeneity along the dendritic tree, with weaker bAP attenuation leading to larger homogeneous portions of the tree near the soma (Fig. 5f). Increasing bAP frequency controls the degree of homogeneity of the proximal tree (Supplementary Fig. 10). Consistent with experimental reports^9^, our model does not produce global organization of receptive field offsets, which are randomly distributed for synapses along the entire dendritic tree (Fig. 5g and Supplementary Fig. 9).

Different degrees of heterogeneity of synaptic orientation preference have been reported in the ferret visual cortex^7^, where experiments distinguished between homogeneous branches, with a mean circular dispersion below 15°, and heterogeneous branches, with a mean circular dispersion above 30°. We compared the cumulative distribution functions of the homogeneous and the heterogeneous branches to proximal (less than two times the attenuation factor) and distal (more than two times the attenuation factor) synapses in our model, respectively, and found good quantitative agreement (Fig. 5h). Computing the distribution of circular dispersions of spines on branches with mean circular dispersion between 15° and 30° in our model revealed branches with intermediate heterogeneity (Fig. 5i). While from the experiments it is not clear whether homogeneous (heterogeneous) branches tend to be more proximal (distal) to the soma, our results predict the global organization of orientation selectivity and suggest that bAP attenuation in different neurons may underlie branch orientation heterogeneity.

In summary, the same modeling framework that produces local orientation clustering of synaptic inputs predicts global synaptic organization on a morphologically realistic model of a layer 2/3 pyramidal neuron with synapses on proximal dendrites sharing similar orientation preference with the soma. We suggest that an attenuating somatic signal is the main factor behind homogenizing some branches of the tree to the same orientation preference as the soma while leaving other branches more heterogeneous and uncorrelated to the soma as observed in the ferret visual cortex^7^.

### Backpropagating action potentials establish visual topography of receptive field centers on mouse dendrites

**Fig. 6:**
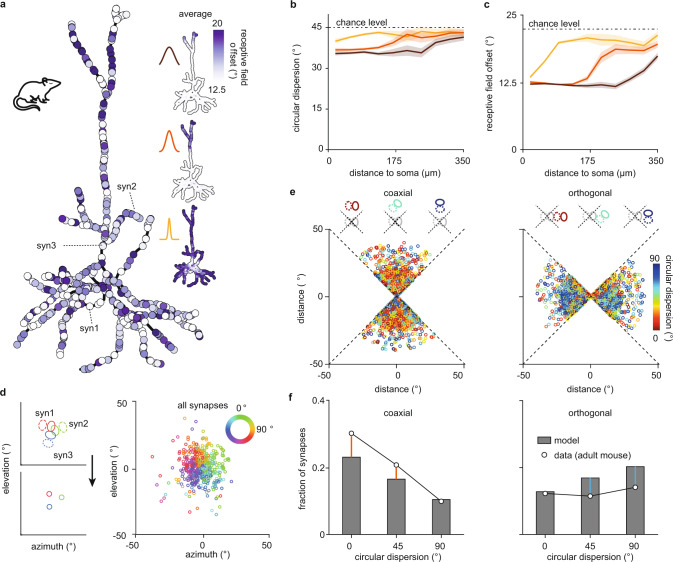
Backpropagating action potentials establish a dendritic map of visual space in a model of the mouse visual cortex. **a** Receptive field offset on the reconstructed pyramidal cell with synapses with a large receptive field center spread corresponding to mouse. Color of synapses indicates the receptive field offset of the associated receptive field. Inset shows the receptive field offset averaged over 80 simulations for the three different attenuation factors (25, 75, and 125 μm) in Fig. 5b. **b**, **c** Circular dispersion (**b**) and receptive field offset (**c**) for the three different bAP attenuation factors in Fig. 5b and as a function of path distance from the soma. Shaded areas indicate 95% confidence interval around the mean. **d** (Left) Illustration of three synaptic receptive fields (see **a**) in visual space (top) and the same receptive fields represented as circles with color denoting orientation preference (bottom). (Right) All synaptic receptive fields from one simulation. **e** Receptive field centers in coaxial (left) and orthogonal (right) visual space colored according to their circular dispersion averaged over 80 simulations. Preferred orientation of the soma is vertical. **f** Histogram of circular dispersions between synaptic and somatic receptive fields in coaxial (left) and orthogonal (right) visual space in the model and experiments (data from adult mouse visual cortex reproduced from ref. ^8^). Preferred orientation of the soma in each schematic is always vertical, as indicated by a gray somatic receptive field in the center, while the preferred orientation of a synapse varies, as indicated by a colored receptive field either in coaxial or orthogonal space (see **e**).

To study emergent global organization in our model of a mouse pyramidal neuron, we considered a larger receptive field diameter and center spread as measured in the mouse (Fig. 4a) and investigated the influence of a somatic bAP signal. Synaptic inputs on the reconstructed pyramidal neuron do not exhibit local orientation clustering, nor global homogenization of orientation preference except for a small bias, as observed experimentally^8,19,20,44^ (Fig. 6b and Supplementary Fig. 9). Synaptic inputs, however, do exhibit local overlap clustering as without a bAP, as well as global organization on the dendritic tree in the presence of a bAP, with synapses close to the soma having overlapping receptive fields with many other synapses close to the soma (Supplementary Fig. 9). As expected from the role of bAPs in reinforcing potentiation and depression near the soma (Supplementary Fig. 8), bAPs homogenize receptive field overlap close to the soma where the bAP influence is the strongest.

In the presence of a bAP, proximal synapses stabilize only if their receptive fields overlap with the somatic receptive field constraining their resulting receptive field offset to be small. Hence, the bAP brings receptive fields of proximal synapses to be on average centered closer to the somatic receptive field compared to the receptive fields of distal synapses (Fig. 6a, c). Distal synapses do not have this constraint, resulting in large cell-to-cell variability at distal branches (Supplementary Fig. 9). This relationship is modulated by bAP attenuation, with weak attenuation increasing the number of synapses centered near the somatic receptive field (Fig. 6c). We refer to this global organization as a dendritic map of visual space. Indeed, in pyramidal neurons of the mouse visual cortex, proximal vs. distal synapses respectively have a tendency to respond to more central vs. peripheral regions of visual space relative to the somatic receptive field center^8,50^. This result is in contrast to the ferret^9^; also in our model, a bAP does not generate a dendritic map of visual space (Fig. 5g) because receptive fields of synaptic inputs proximal to the soma already overlap significantly due to their similar orientation preference to the soma. Hence, shifting their centers toward the somatic center does not increase their overlap and correlation as much as changing their orientation preference (Fig. 4h).

As a result of the global organization of receptive field overlap and dendritic maps generated in our model with bAPs of mouse visual cortex, synapses with nearby receptive field centers in visual space have a similarly oriented receptive field (Fig. 6d). We consequently observed that the distribution of receptive field orientations in different regions of visual space generated in the model has an interesting structure. In particular, synaptic receptive fields positioned along or close to the axis of the somatic receptive field, i.e., in the coaxial space^8,51^, appear to have a similar orientation to the soma (Fig. 6e, left). This mirrors the distribution of circular dispersions in the coaxial region of visual space in the mouse visual cortex^8^ (Fig. 6f, left). The functionally specific organization in coaxial space agrees with the overrepresentation of edges with the same orientation along a common axis found in natural images called colinearity and could be used for the detection of elongated edges^8,52,53^. Interestingly, in our model, synapses in the remaining region of visual space, called the orthogonal space^8^, are more likely to be oriented orthogonally to the soma (Fig. 6e, right). This overrepresentation of large circular dispersions compared to experimental data^8^ (Fig. 6f, right) is, however, consistent with another property found in natural images, called cocircularity, where two segments with different orientation have a tendency to be tangent to the same circle, and which could be used for the detection of continuous and smooth object boundaries^52,53^. We believe that the discrepancy between experiments^8^ and our model might stem from undersampling in the experiments or from additional pruning of connections between cells with large orientation differences after eye opening^54^. These results suggest that, even before the onset of vision, spontaneous activity contains sufficient spatiotemporal information to organize synaptic receptive fields into the orthogonal and coaxial portions of visual space for the detection of elongated edges or continuous boundaries in natural images. This has strong implications for the computational strategies of these neurons and their integration into local microcircuits.

Taken together, our model generates global, in addition to local, organization of synaptic inputs on the dendrites of cortical neurons in the mouse for different features than in the ferret by only including anatomical differences in their visual cortices in terms of the spread and diameter of synaptic receptive fields. In our framework, both the emergence of a retinotopic gradient along the dendritic tree, as well as the accumulation of co-aligned (orthogonally-oriented) synapses in the coaxial (orthogonal) portion of visual space, can be explained through the homogenizing influence of bAPs. Therefore, our model explains the emergence of synaptic organization across scales and in two different species with respect to different stimulus features.

## Discussion

Dendritic compartmentalization of synaptic inputs achieved by clustering has been postulated to enhance the computational capacity of neurons^37^. Yet, direct experimental evidence of clustering during development^4–6,10^ and with respect to different stimulus features in different species in the adult^8,9,12^ has only recently emerged. At the same time, recent work has revealed disparate results regarding the global order of synaptic inputs on the entire dendritic tree^8,9^. To reconcile these findings, we developed a computational framework supported by a specific biophysical implementation based on neurotrophins for the emergence of both local and global synaptic input organization on cortical dendrites in two different species. Combining developmentally inspired synaptic plasticity with retinal wave input and species-specific features of synaptic receptive fields, our framework supports the establishment of local functional clustering for orientation in the adult ferret vs. receptive field overlap in the adult mouse visual cortex^8,9,12^. Including an attenuating somatic signal generates global organization of different features in the ferret and mouse cortex, establishing soma-to-dendrite maps of orientation selectivity and visual topography, respectively. We found that the interaction between two parameters, the cortical magnification factor of visual space and the receptive field diameter, drives these species-specific differences. Interestingly, these parameters can also explain the population-level (columnar vs. salt-and-pepper) organization in the ferret vs. mouse visual cortex^45^, indicating that the same universal developmental process modulated by variations of cortex and retina size can produce both dendritic and population-level organization.

### Relationship to previous modeling studies on clustering

While our work focuses on the establishment of stimulus feature tuning and its organization, previous modeling studies investigated other aspects of synaptic clustering. These include the robust and efficient encoding of memories through activity-dependent plasticity operating over hours^37,38^, the increase of a cell’s computational capacity^55^, and the linking of multiple memories across extended periods^56^. An alternative normative approach proposed the generation of synaptic clusters as the Bayes-optimal solution to a classical conditioning task with unreliable synaptic transmission^57^. However, this model lacked a mechanism for clustering of correlated inputs from different axons. To our knowledge, our framework is the first to explain the emergence of activity-dependent synaptic organization of different stimulus features in a developmental setting and relate it to that in the adult.

### Generality of our modeling framework

Since neurotrophins, calcium, and MMP9 have been implicated in the emergence of functional synaptic clustering during development^6,10^, we based our model for clustering on interactions between these molecules, although additional signaling pathways are likely to be involved in synaptic organization^58–60^. A generalization of this model that derives directly from neurotrophin interactions can be flexibly implemented to apply to other signaling molecules. Possible alternative mechanisms include synaptic tag-and-capture, where plasticity-related-proteins mediate activity-dependent cooperation and competition^28–30^, synaptic crosstalk through the interaction of Rho-family GTPase-mediated proteins, which are in fact modulated by BDNF and involved in structural plasticity^31,32^, or the NMDAR-mediated amplification of calcium signals^21,34^. For the local heterosynaptic depression of unstimulated synapses, alternatives to the neurotrophin mechanism include the activation of calcineurin, IP3Rs, and group I mGluRs^26^ or Arc-targeting of inactive synapses^50,61^. Similarly, the functional roles of MMP9 and calcium could equivalently be filled by postsynaptic depolarization, NMDAR activation, endocannabinoid or nitric oxide signaling, or by more complex diffusible plasticity-related products^62^. Thus, our predictions regarding the organization of synapses are not contingent on a specific biophysical implementation of our model, as long as it implements distance- and timing-dependent competition between synapses, and integrates slow developmental activity patterns matched to the timescales of plasticity.

### Origin of clustered synaptic input

A prominent source of excitatory inputs to layer 2/3 pyramidal neurons comes from layer 4 neurons^51^, which likely obtain their orientation selectivity by combining On and Off center-surround receptive fields of thalamic feedforward inputs^63^. Our modeling framework also supports the concurrent emergence and clustering of orientation selectivity in the cortex through the clustering of On and Off thalamocortical synapses (Supplementary Fig. 11). Also, pyramidal cells in the mouse and ferret visual cortex are already orientation-selective at^54^ and before^64,65^ eye opening, providing another likely source of the synaptic Gabor receptive fields in our model.

### Relationship between developing and mature cortex

Several factors make our model appropriate for the establishment of synaptic organization in development: the high turnover of synapses, the ability of synaptic inputs to interact over long distances, and the slow timescale of spontaneous activity and neurotrophin interactions. Therefore, in this developmental setting, STDP rules that operate on fast timescales involving several milliseconds do not generate clustering (Supplementary Fig. 5), in agreement with other studies of developmental plasticity^24,27,33^. While early development is a particularly opportune time for the emergence of synaptic organization^4–6,10^, it is currently unknown how clusters formed during development relate to those observed in the adult animal. Given the continued turnover of synapses in the adult brain^43^ and the formation of new clusters following altered sensory experience^58^ and learning^59,60^, it is a priori unclear whether the clusters formed during early development persist into adulthood. Our developmentally inspired model for synaptic competition combined with correlated activity from retinal waves is sufficient to produce functional and feature-specific clustering as in the adult and can provide a backbone for clustering even when the density of synapses increases dramatically. Already-established clusters in our model can be preserved by decreasing the postsynaptic spread in calcium^21^ (Supplementary Fig. 12), although other changes (such as the onset of inhibition, postsynaptic thresholding through a nonlinearity, or a decrease in proBDNF level) would be equally suited. This supports an interpretation where development equips dendrites with basic building blocks such as feature selectivity from which other functional properties are derived in adulthood. It would be interesting to investigate whether context-specific clustering as in the retrosplenial^60^ or the motor^59^ cortex occur alongside (rather than in competition with) clusters formed during development, as described in the auditory system of the juvenile barnowl^58^, and how our proposed plasticity mechanism might interact with STDP rules relevant in the adult^66^.

### Functional role of clustered synapses

We propose three situations where synaptic input clustering may be beneficial for a neuron. (1) The transient, precise synchronization of even a small group of synapses (the exact number of synapses required in vivo is unknown^34^) can result in the nonlinear summation of synaptic activity^67^, enhancing a neuron’s computational capacity^37^. These nonlinearities can furthermore counteract location-dependent gradients of conductances across synapses, effectively establishing a synaptic democracy^11,68^. (2) Since synaptic transmission is highly variable^69^, multiple synapses encoding a similar signal (in combination with local supralinear integration) increase tolerance for different types of noise, some of which cannot be removed by averaging^70^. (3) Furthermore, since the translation of proteins is localized to individual dendritic compartments where nearby synapses share available proteins, synaptic clustering is also beneficial from the perspective of resource-preservation, consistent with the sharing of plasticity-related proteins in models like synaptic tag-and-capture^29,71,72^.

### Inhibitory synapses could form a backbone for excitatory clustering

Our modeling framework focused on the emergence of fine-scale organization of excitatory (glutamatergic) inputs, primarily due to lack of experimental data on the role of inhibitory (GABAergic) synapses on clustering. We speculate, however, that inhibitory synapses might shape the clustering of excitatory synapses on the dendritic tree. Indeed, while not being clustered themselves, GABAergic synapses can constrain the orientation preference of nearby excitatory clusters in our model (Supplementary Fig. 13). Since GABA might be excitatory early in postnatal development^73,74^, we considered two scenarios in our model with GABA switching from excitatory to inhibitory or being inhibitory the entire time. The resulting excitatory clusters are tuned to the same orientation relative to nearby inhibitory synapses in the former case or tend to prefer the orthogonal orientation in the latter case (Supplementary Fig. 13). Thus, we predict that GABAergic synapses might be co-clustered with excitatory synapses. Provided that GABAergic synapses fire in synchrony with glutamatergic synapses, this co-clustering could allow them to dynamically switch a given cluster on or off^75^. Additionally, since inhibitory synapses can cancel the effect of backpropagating action potentials^76^, we expect that inhibitory synapses synchronized with somatic activation would be able to protect a cluster from the homogenizing effect of backpropagating somatic signals^77^.

In summary, starting from several ingredients, including a species-specific receptive field model based on anatomical considerations, retinal wave input, and a developmentally inspired plasticity based on neurotrophins, our modeling framework generates species-specific outcomes regarding the emergence of local and global organization of dendritic synaptic inputs. These outcomes combine several experimental studies from the last decade on the emergence of functional synaptic organization across scales, and with respect to different stimulus features in two species. Therefore, our framework can explain how circuits wire up with subcellular precision, with paramount implications on the computational properties of cortical neurons and networks.

## Methods

### Neurotrophin model

We based the neurotrophin plasticity model on interactions between signaling molecules shown to drive the emergence of synaptic clustering during development^4–6,10^: BDNF $({\bf{B}})$, its immature form, proBDNF (**P**), the cleaving protease MMP9 (**M**), and postsynaptic calcium (**Y**). W_*k*_ is the synaptic efficacy of a synapse with hard bounds at zero and one, and initial efficacy of 0.5. For a pair of synapses *k* and *l* separated by ${d}_{kl}$ along the branch, we defined the proximity variables ${s}_{kl}={e}^{-\frac{{d}_{{kl}}^{2}}{2{\sigma }_{s}^{2}}}$, where *σ*_*s*_ determines the spatial postsynaptic calcium spread constant.

Since the mechanism that produces synaptic clusters is activity-dependent, we modeled presynaptic and postsynaptic accumulators of synaptic activity. We modeled the presynaptic accumulator MMP9 as a synapse-specific leaky accumulator^22^ with dynamics

2 $${\tau }_{M}\frac{d{M}_{k} }{dt}=-{M}_{k} (t)+\phi {x}_{k} (t)$$

Here,

3 $${x}_{k}(t)=\int_{0}^{\infty }{\sum }_{f}\delta (s-{s}_{k}^{f})(H(t-s)-H(t-{x}_{{\rm{dur}}}-s))ds$$

is the input event train to the *k*-th synapse with events at times ${t}_{k}^{f}$ and where the Heaviside step function $H(t)$ is 0 when *t* is less than 0 and 1 when *t* is greater than or equal 0, so that events have duration ${x}_{{\rm{dur}}}$. $\phi$ is an MMP9 efficiency constant that determines how efficiently MMP9 converts proBDNF into BDNF per unit of time. The postsynaptic accumulator was modeled by the local calcium variable **Y** that integrates activity from nearby synapses^21^, weighted by their efficacies $W_{l}$ and the distance-dependent factor ${s}_{{kl}}$,

4 $${\tau }_{Y}\frac{d{Y}_{k}}{dt}=-{Y}_{k}(t)+\mathop{\sum }\limits_{l=1}^{N}{s}_{kl}{W}_{l}(t){x}_{l}(t)$$

The lateral spread of calcium might be passive (during development many excitatory synapses form on the dendritic shaft^78^ where calcium is less compartmentalized) or active (such as the calcium-induced calcium release characterized in the developing hippocampus^21^). Following experimental data^10,31^, we coupled extracellular proBDNF and BDNF to postsynaptic calcium and let MMP9 convert proBDNF to an equal amount of BDNF,

5 $${\tau }_{P}\frac{{{dP}}_{{{k}} }}{{dt}}{=-P}_{{{k}} }(t)+(1-\eta )Y(t){\;-\;M}_{{{k}} }(t){P}_{{{k}} }(t)$$

6 $${\tau }_{B}\frac{d{B}_{{{k}} }}{{dt}}=-{B}_{{{k}} }(t)+\eta Y(t)+{M}_{{{k}} }(t){P}_{{{k}} }(t).$$

Here, the scaling factors $(1-\eta )$ and *η* ensure that without MMP9 the ratio of BDNF to proBDNF remains close to the constitutive ratio *η*^25^ which is dominated by proBDNF (Table 1). By coupling the neurotrophin level to the local calcium level, inactive synapses experience an increase in extracellular neurotrophin following the activation of nearby activated synapses. While there is indirect evidence that activity-dependent BDNF release is not restricted to the activated synapse^79^ and that stimulation of a synapse increases BDNF-receptor activation at another synapse^31^, much less is known about the activity-dependent release of proBDNF. For our model, we assumed that both BDNF and proBDNF release depend only on an influx of postsynaptic calcium and can therefore also occur at unstimulated synapses when other nearby synapses are stimulated. According to the Yin-Yang hypothesis of neurotrophin action^15^, binding of BDNF to its receptor TrkB (tropomyosin receptor kinase B) leads to synaptic potentiation, while binding of proBDNF to p75^NTR^ receptor leads to depression. We assumed that the binding affinities of BDNF (α) and proBDNF (*β*), and corresponding magnitudes of induced plasticity are balanced, so that the synaptic efficacy can be written as the difference between BDNF and proBDNF,

7 $${\tau }_{W}\frac{d{W}_{{{k}}}}{{dt}}=\alpha {B}_{{{k}}}(t)-\beta {P}_{{{k}}}(t),$$

with *α* = *β* = 1 for all simulations except in Supplementary Fig. 3. *τ* always denotes the time constant for the variable in the corresponding subscript. Despite the model’s biophysical motivation, in agreement with other modeling studies,^56,80^ all modeled variables are unitless, therefore, they are to be interpreted relative to each other.

### Generalized neurotrophin-inspired model

We reduced the above neurotrophin model to a generalized model for clustering which is analytically tractable, assuming a tight coupling of proBDNF and BDNF to the postsynaptic calcium. We note, however, that this assumption is not critical (see Supplementary Fig. 1). We made a steady-state approximation of ${P}_{{{k}} }$ and ${B}_{{{k}} }$ in Eqs. (5) and (6), inserted these expressions into Eq. (7) for ${W}_{{{k}}}$ and then linearized the resulting function around ${M}_{{{k}}}=0$ to obtain the generalized model (see Supplementary Note 1 for details). We note that this linearization strips away some higher-order terms that have an attenuating effect on MMP9 in the full model (Supplementary Note 1). Hence, the effect of the presynaptic accumulation (a proxy for MMP9) in the generalized model is slightly amplified, shifting the system into a more potentiation driven regime. As a consequence, the generalized model experiences slightly less depression and competition compared to the full neurotrophin model, which results in fewer synaptic turnovers and slightly weaker clustering of synapses (Supplementary Fig. 2).

While we used upper case letters for the variables in the full neurotrophin model, we used lower case letters for the generalized model. The model consists of a synapse-specific presynaptic accumulator ${v}_{k}$ (from now on we use the dot notation to denote the derivative, ${\dot{v}}_{k}=\frac{d{v}_{k}}{{dt}}$),

8 $${\tau }_{v}{\dot{v}}_{k}=-{v}_{k}(t)+\phi {x}_{k}(t),$$

and a postsynaptic accumulator ${u}_{k}$ that averages over nearby synapses in a weighted and distance-dependent manner,

9 $${\tau }_{u}{\dot{u}}_{k}=-{u}_{k}(t)+\mathop{\sum }\limits_{l=1}^{N}{s}_{{kl}}{w}_{l}(t){x}_{l}(t).$$

The efficacy equation (Eq. 7) turns into a Hebbian equation that directly combines the pre- and postsynaptic accumulator with an additional offset constant *ρ*,

10 $${\tau }_{w}{\dot{w}}_{k}={u}_{k}(t)({v}_{k}(t)+\rho ),$$

with $\rho =\frac{(\alpha\,+\,\beta )\eta\,-\,\beta }{(\alpha\,+\,\beta )(1\,-\,\eta )}$ and ${\tau }_{w}={\tau }_{W}\frac{1}{(\alpha\,+\,\beta )(1\,-\,\eta )}$. This model cannot be reduced further without losing either the dependence on correlation through the link to the BTDP rule, or the dependence on distance.

### Steady-state analysis of the generalized neurotrophin-inspired model

Combining the equations for the accumulators and the efficacy dynamics (Eqs. 8–10), taking the expected value over neurons (denoted by 〈⋅〉) and over time (denoted by ${{\rm{lim}}}_{T\to \infty }\frac{1}{T}{\int }_{0}^{T}\cdot {dt}$), we can write the expected change in synaptic efficacy as (see Supplementary Note 2 for full derivation)

11 $$\begin{array}{c}\mathop{{\rm{lim}}}\limits_{T\to \infty }\frac{{\tau }_{w}}{T}{\int }_{0}^{T}\langle {\dot{w}}_{k}\rangle {dt}=\phi \mathop{\sum}\limits_{l}{s}_{{kl}}{w}_{l}\left({\int }_{-\infty }^{\infty }{\bar{\gamma }}_{{kl}}(s)\varGamma (s){ds}+{\mu }_{k}{\mu }_{l}\right)+\rho \mathop{\sum}\limits_{l}{s}_{{kl}}{w}_{l}{\mu }_{l}.\end{array}$$

Here, ${\mu }_{k}={{\lim}}_{T\to {\infty }}\frac{1}{T}{\int }_{0}^{T}{x}_{k}(t){dt}$ denotes the mean firing rate of the *k*-th input $x_{k}$, ${\bar{\gamma }}_{{kl}}(t)={{\lim}}_{T\to {\infty }}\frac{1}{T}{\int }_{0}^{T}({x}_{k}(s)-{\mu }_{k})({x}_{l}(s-t)-{\mu }_{l}){ds}$ denotes the covariance between inputs *k* and *l* at lag *t* and the kernel is given by $\varGamma (t)=\frac{1}{{\tau }_{u}+{\tau }_{v}}{e}^{-\frac{{\rm{|}}t{\rm{|}}}{{\tau }_{u}}}.$

When only one input is activated with burst events of duration ${x}_{{\rm{dur}}}$ and rate $\mu$, we simplified Eq. (11) to write the change in synaptic efficacy for this input as $\langle {\dot{w}}_{1}\rangle ={K}_{1}\mu +{K}_{2}{\mu }^{2}$, while the change in efficacy for a second inactive input at a distance ${d}_{12}$ is $\langle {\dot{w}}_{2}\rangle ={K}_{3}\mu {e}^{-\frac{{d}_{12}^{2}}{2{\sigma }_{s}^{2}}}$, with constants ${K}_{1},{K}_{2}$, and ${K}_{3}$ (see Supplementary Note 3 for details), as shown in Fig. 1d.

We considered the case with identical inputs on a linear dendrite with density $\nu$, equal efficacies ${w}_{k}=w$ and rates ${\mu }_{k}=\mu$ for all $k$, and identical correlation ${c}_{{{{kl}}}}=c$ for all pairs $k\,\ne\,l$. In this setting, from Eq. (11) we derived the critical correlation ${c}{\ast }$ at which the system switches from the depression-dominated into the potentiation-dominated regime, $\langle{{\dot{w}}\rangle ({c}{\ast })=0}$, as ${c}{\ast }=\frac{\kappa S-1}{S-1}$ where ${S}_{{{k}}}=\mathop{\sum}_{l}{s}_{{kl}}\approx \sqrt{2\pi }{\sigma }_{c}\nu$, which is the same for all inputs $k$, thus $S={S}_{{{k}}}$, and $\kappa =(-\frac{\rho }{\phi }-\mu )({\tau }_{u}+{\tau }_{v})$ is a constant (see Supplementary Note 4). $S$ can be thought of as a measure of the total amount of activity in an area around a given synapse. Note that for high densities $\nu$, the critical value ${c}{\ast }$ quickly approaches $\kappa$ and is bounded above by it. The expression for ${c}{\ast }$ determines the dashed line in Fig. 2b, while the other contour lines come directly from Eq. (11) and the approximation $S\approx \sqrt{2\pi }{\sigma }_{c}\nu$ (see Supplementary Fig. 14 and Supplementary Note 4). Whether a synapse becomes stabilized or depressed is thus determined by the balance of two factors: (1) A homosynaptic component, which depends only on the activation of the given synapse and is always stabilizing (Fig. 1d, top); (2) A heterosynaptic component, which depends only on the activation of synapses in the neighborhood of the activated synapse and can be either depressing or stabilizing. A synapse becomes depressed when the heterosynaptic component is depressing and outweighs the homosynaptic component. We first determined the correlation beyond which there is no competition between synapses, $\kappa =\left(-\frac{\rho }{\phi }-\mu \right)\left({\tau }_{u}+{\tau }_{v}\right)\approx 0.32$, due to a stabilizing, rather than depressing, heterosynaptic component (see Supplementary Note 4 for details). Note that this parameter depends on the proBDNF to BDNF constitutive ratio, $\eta$ (through $\rho$), on the MMP9 efficiency constant, $\phi$, the stimulation rate of the synapse, *μ*, and the time constants of the pre- and postsynaptic accumulators in the model. Second, we found that when the heterosynaptic component is depressing for correlations below $\kappa \approx 0.32$, the synaptic density, $\nu$, plays an additional role in determining whether the depressing heterosynaptic component outweighs the stabilizing homosynaptic component.

### Parameters and data fitting

Many of the model parameters were extracted from published experimental data as follows.

Experimental work has shown that the binding affinity of BDNF to TrkB and proBDNF to p75^NTR^ is very high (see ref. ^81^ and references therein). Therefore, we assumed that the decay time constants of proBDNF and BDNF are small and, in the absence of evidence otherwise, equal (${\tau }_{P}={\tau }_{B}$). In our model, the decay time constants of proBDNF and BDNF describe the tightness of coupling between the level of postsynaptic calcium and of free (unbound) extracellular neurotrophin. This produces much longer effective decay time constants ${\hat{\tau }}_{B}\approx {\hat{\tau }}_{P}$, as measured in ref. ^31^ (Supplementary Fig. 15).

The synaptic efficacy time constant ${\tau }_{W}$ was chosen so that the total change in synaptic efficacy integrates the contribution of multiple pre- and postsynaptic events, yielding slow weight change compared to much faster neural activity, consistent with other computational models of synaptic plasticity^82^. The value for the time constant of postsynaptic calcium decay, ${\tau }_{Y}$ (300 ms), is in the experimentally measured range between 200–700 ms^21^. We based the decay time constant of extracellular MMP9 (${\tau }_{M}$) on a recent study which measured the decay of MMP9-GFP signal of a single exocytosis event in MCF-7 cells^83^.

We assumed that the constitutive percentage of BDNF $(\eta)$ of the total released neurotrophin is $\eta =45 \%$ based on estimates in the hippocampus^84^, although it is possible that this ratio can change across different brain regions, over the course of development and depending on the stimulation protocol. Our sensitivity analysis (Supplementary Fig. 1) and perturbation simulations (Supplementary Fig. 3) demonstrate that the constitutive percentage of BDNF $(\eta )$ is crucial in regulating the overall amount of potentiation and depression of synaptic efficacy, and therefore has a strong impact on the emergence and maintenance of synaptic organization. Therefore, although in our model stimulation of a given synapse leads to its potentiation consistent with some experimental data^31^ (Fig. 1), decreasing $\eta$ could lead to the depression of the stimulated synapse in agreement with other experimental data^6^.

The MMP9 efficacy constant ($\phi$) interacts closely with the constitutive percentage of BDNF ($\eta$). This relationship becomes clear in the generalized neurotrophin-inspired model, where $\phi$ and $\eta$ trade off to maintain a given value of the critical correlation ${c}{\ast }$ and ensure the selective competition between strongly and poorly synchronized synapses. Since ${c}{\ast }$ depends on $\kappa =(-\rho /\phi -\mu )({\tau }_{u}+{\tau }_{v})$, $\phi$ needs to be proportional to $\rho =\frac{2\eta -1}{2(1-\eta )}$. We used $\phi =\frac{3}{50}$ ms^−1^ to obtain ${c}{\ast }$ with appropriate synaptic competition (Fig. 2b) given experimentally measured synaptic density. In particular, the density of synapses in the developing sensory cortex increases from $0.2$ ${\rm{\mu }}{{\rm{m}}}^{-1}$ to $0.8\!-\!\!1.2$ ${\rm{\mu }}{{\rm{m}}}^{-1}$ during postnatal development^85,86^. We used a fixed density of $0.2$ ${\rm{\mu }}{{\rm{m}}}^{-1}$ for computational tractability as the runtime of our simulations scales as ${\mathscr{O}}{({N}^{2})}$ in the number of synapses $N$. However, we also found that synaptic clustering is not perturbed when synaptic density increases during simulated development (Supplementary Fig. 12). Here, we increased the density of synapses from $0.2$ ${\rm{\mu }}{{\rm{m}}}^{-1}$ to $0.8$ ${\rm{\mu }}{{\rm{m}}}^{-1}$ ^85^ within four days, by adding a new synapse at a random position and with random receptive field orientation at regular time intervals of 64 min. Additionally, to conserve the total amount of postsynaptic calcium, we decreased the amount of calcium spread and the amount of released calcium per synaptic event with increasing time^21^.

To determine the standard deviation of calcium spread, ${\sigma }_{c}$, we referred to experimental studies that report mean propagation distances (defined as the full width at half maximum, FWHM) from individual synapses in dendrites of a developing pyramidal neuron, reporting values between $7.97$ ${\rm{\mu }}{\rm{m}}$^21^ and $17.6$ ${\rm{\mu }}{\rm{m}}$^4^. Assuming a Gaussian spread profile, these FWHM values translate into a standard deviation between $3.4$ ${\rm{\mu }}{\rm{m}}$ and $7.5$ ${\rm{\mu }}{\rm{m}}$, which validates our choice of ${\sigma }_{c}=6$ ${\rm{\mu }}{\rm{m}}$.

The values for all parameters used in our simulations are listed in Table 1.

### Sensitivity analysis

We performed a sensitivity analysis to determine the sensitivity of the central building blocks of our model, the shape of the resulting BTDP rule (Fig. 1), and the emergence of local clustering (Fig. 3), to perturbations of different model parameters. To quantify how uncertainty in the input parameters propagates, we defined distributions over our model parameters that were chosen to be sufficiently broad to sometimes produce poor clustering (Supplementary Fig. 1). Many of the distributions were truncated from below because choosing extremely small values of any parameter is not biologically plausible and cannot be implemented in the model—but the distributions still cover one order of magnitude relative to the nominal parameter value. The prior distributions used for the sensitivity analysis are: ${\tau }_{M},{\tau }_{v}\;{{\sim }}{\mathscr{N}}(600,300)$ ms; truncated from below at 5 ms, ${\tau }_{Y},{\tau }_{u}\;{{\sim }}{\mathscr{N}}(300,150)$ ms; truncated from below at 5 ms, ${\tau }_{B},{\tau }_{P}\sim {\rm{exp }}\left({\mathscr{N}}\left(4,1\right)\right)$ ms; truncated from below at 1 ms, ${\sigma }_{c}{\mathscr{\sim }}{\mathscr{N}}\left(8,1\right)$ ${\rm{\mu }}{\rm{m}}$; truncated from below at $4$ ${\rm{\mu }}{\rm{m}}$, $\phi \sim {\mathscr{N}}(\frac{3}{50},\,\frac{3}{500})$, $\eta \sim {\mathrm{Uniform}}([40 \% ,50 \% ])$.

### Retinal wave generation

To generate retinal waves with realistic spatiotemporal properties, we simulated 6 h of retinal waves from a published computational model with the parameter setting for mice (P0–P13)^41^ and looped the waves over the entire duration of the simulation. For consistency, we used the mouse retinal wave parameter settings for all simulations, since the parameter settings for ferret come from much younger animals (P2–P4)^41^. As a control we also generated white noise input by sampling each pixel independently from a normal distribution and applying a spatial Gaussian filter with a 2° standard deviation. Subsequently, we used each frame of the retinal wave or the white noise movie as input to a linear-nonlinear Poisson model of event generation.

### Linear-nonlinear model

The linear filter consists of a Gabor linear filter ${\bf{H}}$, which we split into two components, a positive and a negative Gaussian with the same shape and opposite sign. We chose the semi-minor axis to be half as large as the semi-major axis so that the resulting Gabor is approximately equal in diameter along all axes. The receptive field diameter depends on the species and was extracted from published literature (Table 1). Synaptic receptive field centers in visual space have been measured to spread out in a small (for ferret^9^) or large (for mouse^8,51^) neighborhood around the somatic receptive field center. The spread of the receptive field centers is inversely proportional to the size of the corresponding visual cortices, so that for the ferret visual cortex which is five times the size of the mouse visual cortex, the spread of receptive field centers in the ferret is one fifth the spread in the mouse. We interpret this anatomical argument to mean that the product of the receptive field spread and the diameter of the visual cortex is constant for the two species. For macaque we then use this relationship to infer the receptive field spread from the experimentally measured visual cortex diameter to obtain 2°. We defined the receptive field center spread ${\sigma }_{p}$ to incorporate these differences and estimated ${\sigma }_{p}$ from extracted experimental data in both species^8,9^ as the standard deviation of a Gaussian (${a}_{0}{\rm{exp }}(-({x}^{2}/(2{\sigma }_{p}^{2})))$) (Fig. 4a–c and Table 1). A synaptic receptive field center was drawn from a two-dimensional symmetric Gaussian with standard deviation ${\sigma }_{p}$ and truncated to a circle of radius 50° to ensure that receptive fields fall within a region of visual space where they are modulated by the retinal waves. Each individual Gabor filter is rotated according to its orientation, $\theta$, between 0° and 360°. For the simulations in Supplementary Fig. 11, we modeled On- and Off-selective receptive fields as Gaussians with a positive or negative sign and a diameter that is matched to typical mouse LGN neurons^87^. The linearly filtered stimulus is passed through an exponential nonlinearity, $a\,{\rm{exp }}(b\,H)$, that produces an instantaneous firing rate from which we generate a Poisson input train with individual 50 ms-long events. The parameters ($a,b$) were chosen ($a=0.2$ Hz, $b=9.4$) to achieve a burst rate of around 15 min^−1^, as found experimentally^4^. For the simulations in Supplementary Fig. 11 we set the spontaneous background firing rate of Off-selective inputs to twice the value of On-selective inputs^88^, $a=$ 0.4 Hz.

### Structural plasticity

To model synaptic turnover, we implemented a structural plasticity rule inspired by ref. ^43^ where each synapse whose efficacy falls below a fixed threshold ${W}_{{\rm{thr}}}$ is removed and replaced by a new synapse with a random position on the branch and a randomly oriented Gabor receptive field (Fig. 3d). The newly generated efficacy of a synapse through structural plasticity is the same as the initial efficacy at the onset of the simulation, i.e. $0.5$, and the receptive field has orientation drawn from a uniform distribution over the interval [0°, 360°]. The turnover threshold is chosen arbitrarily to be sufficiently small to minimize the possibility of accidental removal of a synapse that is well-correlated with its neighbors. A novel synapse can potentially come from a pool of silent synapses, a type of synapse that lacks AMPA receptors but can become unsilenced through activity-dependent mechanisms^89^.

### Dendritic nonlinearity and cooperative plasticity

Inspired by experiments^67^, we used a sigmoidal-like dendritic nonlinearity, $g(I)=\gamma \frac{{c}_{1}}{1+{\rm{exp }}(-{c}_{2}(I-{c}_{3}))}+(1-\gamma )I$ where the parameter $\gamma \in [{0,1}]$ controls the strength of the nonlinearity and ${c}_{1}=0.5,{c}_{2}=35,{c}_{3}=0.125$ (Supplementary Fig. 6). This nonlinearity modifies the equation for the postsynaptic accumulation (Eq. 10),

12 $${{{\tau }}}_{{{u}}}{{\dot{u}}}_{{{k}}}({{t}})=-{{{u}}}_{{{k}}}({{t}})+{{g}}\left(\mathop{\sum }\limits_{{{l}}=1}^{{{N}}}{{{s}}}_{{{kl}}}{{{w}}}_{{{l}}}({{t}}){{{x}}}_{{{l}}}({{t}})\right).$$

The nonlinearity boosts the amount of postsynaptic calcium that is released through the simultaneous activation of multiple nearby synapses. This effectively imposes the constraint that multiple synapses have to be active to generate a postsynaptic calcium event.

Similarly, we investigated whether synaptic organization can emerge when imposing a threshold for cooperativity, as recently described for NMDA-dependent cooperative potentiation^34,90^ and cooperative heterosynaptic depression^26^ adulthood. We adapted Eq. (10) for the change in synaptic efficacy to include a spacing and a timing cooperativity threshold,

13 $${\tau }_{w}{\dot{w}}_{k}(t)={\mathcal{F}}({u}_{k}\left({t}\right)({v}_{k}\left(t\right)+\rho ))$$

where ${\mathcal{F}}(x)=x$ when more than three synapses within a distance from synapse $k$ smaller than a spacing threshold were activated within a timing threshold, and ${\mathcal{F}}(x)=0$ otherwise. We chose three synapses guided by experimental data^90^, but this number can be traded off with the spacing threshold.

### Backpropagating somatic signal

To investigate the emergence of global order of synaptic inputs on an entire dendritic tree, we included a somatic accumulator which can produce backpropagating action potentials (bAPs) which attenuate with distance from the soma. Our somatic accumulator sums linearly over all postsynaptic accumulators, weighing them by their respective synaptic efficacy^37,91^, $A(t)=\mathop{\sum }\nolimits_{k=1}^{N}{w}_{k}(t){u}_{k}(t)$. If the somatic accumulator crosses a threshold, ${A}_{{\rm{th}}}$, there is a 25% probability that a bAP is generated, $B(t)\in \{{0,1}\}$. These two parameters were chosen so that the burst rate of the soma is similar to the firing rate of synapses (15 min^−1^) while avoiding step like activation patterns. The resulting somatic activation thus incorporates some aspects of the burst firing common for developing neurons, where the burst rate is modulated by the probability of generating a bAP (Supplementary Fig. 10). The bAP affects the postsynaptic accumulators of all synapses with attenuating effect over distance^47,48^

14 $${\tau }_{u}{\dot{u}}_{k}(t)=-{u}_{k}(t)+\mathop{\sum }\limits_{l=1}^{N}{s}_{kl}{x}_{l}(t){w}_{l}(t)+{s}_{k}{B}_{\mathrm{amp}}B(t).$$

Here, ${B}_{{\rm{amp}}}$ is the unattenuated strength of the bAP and ${s}_{k}={e}^{-\frac{{d}_{k}^{2}}{2{\sigma }_{s}^{2}}}$ is the attenuation factor of synapse $k$ that depends on the distance to the soma ${d}_{k}$ (Fig. 5b). We chose ${B}_{{{\mathrm{amp}}}}$ (Table 1) to reflect the experimental observation that the calcium influx through a bAP is larger than that caused by synaptic activation^7^. Additionally, a computational model^48^ found that the peak calcium concentration at individual spines is around $29$ μM (independent of distance) in a subthreshold stimulation protocol, while it is between $30$ μM (distal synapse) and $100$ μM (synapse at $150$ μm from the soma). Extrapolating this to a distance of $0$ μm makes a value of 5 for ${B}_{{\rm{amp}}}$ plausible, since this is five times as large as the synaptic level of calcium produced by presynaptic activation. Note that we did not include the term with $B(t)$ in the sum for the somatic accumulator $A(t)$ to avoid a positive feedback loop.

Since bAPs have been found to induce neurotrophin release in a calcium dependent manner^49^, we also considered the case of including a bAP in the neurotrophin model by modifying the equation for the postsynaptic calcium (compare to Eq. 3),

15 $${\tau }_{Y}\frac{d{Y}_{k}}{dt}=-{Y}_{k}(t)+\mathop{\sum }\limits_{l=1}^{N}{s}_{kl}{x}_{l}(t){W}_{l}(t)+{s}_{k}{B}_{\mathrm{amp}}B(t).$$

Placing a synaptic input at increasing distances away from the soma along the dendritic tree of the reconstructed layer 2/3 dendrite, we used either the distance-dependent competition or the burst-timing-dependent plasticity protocol from Fig. 1 to stimulate the soma and synaptic input (Supplementary Fig. 8).

### Inhibitory clustering

To investigate the role of inhibitory synapses in synaptic clustering, we extended our neurotrophin model to also include plastic, GABAergic synapses. While it is commonly accepted that GABAergic synapses are plastic, the nature of the inhibitory plasticity is not well known, strongly depends on the cortical region and can easily be modulated by diverse neuromodulators^92^. During development, BDNF-TrkB signaling at excitatory synapses produces potentiation of nearby GABAergic synapses^93^ and proBDNF-p75^NTR^ signaling results in the depression of nearby GABAergic synapses, provided that the p75^NTR^ activation occurs in concert with the opening of NMDA receptors^94^. Therefore, we assumed that the strength of GABAergic synapses is homeostatically regulated to maintain a balance of excitation and inhibition^95^. We postulated that the plastic change of inhibitory synapses, $\frac{d{W}_{k}^{{\rm{GABA}}}}{{dt}}$, closely follows the average amount of plasticity of nearby excitatory synapses (Supplementary Fig. 13a),

16 $$\begin{array}{c}\begin{array}{c}{{{\tau }}}_{{{W}}}\frac{{{d}}{{{W}}}_{{{k}}}^{{{{GABA}}}}}{{{dt}}}=\mathop{\sum }\limits_{{{l}}=1}^{{{{N}}}_{{{I}}}}{{{s}}}_{{{kl}}}({{{B}}}_{{{l}}}({{t}})-{{{P}}}_{{{l}}}({{t}})),\end{array}\end{array}$$

where the sum runs over all the glutamatergic synapses $l$. Input to the GABAergic synapses, ${x}_{k}^{{\rm{GABA}}}$ and structural turnover of GABAergic synapses is implemented in the same way as for glutamatergic synapses. Since there is an ongoing debate on whether GABA during development is inhibitory^96^, or whether it is initially excitatory and only later switches to being inhibitory^73,74^, we considered two scenarios (Supplementary Fig. 13a):

A. GABAergic synapses hyperpolarize the postsynaptic membrane and are able to decrease postsynaptic calcium^97^,

17 $$\begin{array}{c}\begin{array}{c}{{{\tau }}}_{Y}\frac{{{d}}{{{Y}}}_{{{k}}}}{{{dt}}}=-{{{Y}}}_{{{k}}}({{t}})+\mathop{\sum }\limits_{{{l}}=1}^{{{{N}}}_{{{E}}}}{{{s}}}_{{{kl}}}{{{W}}}_{{{l}}}{{{x}}}_{{{l}}}({{t}})-\mathop{\sum }\limits_{{{l}}=1}^{{{{N}}}_{{{I}}}}{{{s}}}_{{{kl}}}{{{W}}}_{{{l}}}^{{{{GABA}}}}{{{x}}}_{{{l}}}^{{{{GABA}}}}({{t}}),\end{array}\end{array}$$

B. GABAergic synapses are initially excitatory and are able to increase postsynaptic calcium,

18 $$\begin{array}{c}\begin{array}{c}{{{\tau }}}_{{{Y}}}\frac{{{d}}{{{Y}}}_{{{k}}}}{{{dt}}}=-{{{Y}}}_{{{k}}}({{t}})+\mathop{\sum }\limits_{{{l}}=1}^{{{{N}}}_{{{E}}}}{{{s}}}_{{{kl}}}{{{W}}}_{{{l}}}{{{x}}}_{{{l}}}({{t}})+\mathop{\sum }\limits_{{{l}}=1}^{{{{N}}}_{{{I}}}}{{{s}}}_{{{kl}}}{{{W}}}_{{{l}}}^{{{{GABA}}}}{{{x}}}_{{{l}}}^{{{{GABA}}}}({{t}}),\end{array}\end{array}$$

and only later in the simulation switch to being inhibitory and decreasing postsynaptic calcium^73^, see Eq. (17).

Note that in the first scenario we needed to impose the biologically realistic condition ${Y}_{k}\,> \,0$. In both scenarios, we scaled the amount of calcium increase per excitatory synaptic event so that the total amount of calcium in a neighborhood remains constant over time to stay in the same dynamic regime as in the exclusively glutamatergic case. We implemented the switch from excitation to inhibition in the second scenario after 4 days, although the exact day of the switch does not influence our results since the fraction of stable synapses increases at the same speed as in Fig. 3e.

### Simulations

For the simulations in Fig. 1b, d, we distributed two synapses at varying distances ($\varDelta d=0$ ${\rm{\mu }}{\rm{m}}$ to $15$ ${\rm{\mu }}{\rm{m}}$) on a linear dendrite of length $L=150$ ${\rm{\mu }}{\rm{m}}$ with periodic boundary conditions. Only one input was stimulated with a train of continuous bursts of activation whose rate varies from 1 to 20 min^−1^. All bursts in our model have a duration of 50 ms (${x}_{{\rm{dur}}}=$ 50 ms) unless stated otherwise. We fixed the synaptic efficacy to the initial value $W(t)=0.5$ and computed the expected change in the efficacy of synapse $k$ in Eq. (7) as the temporal average of ${B}_{k}(t)-{P}_{k}(t)$ over the 40 min duration of the simulation. For the simulations in Fig. 1f, g, we simulated only one synapse and provided either one (panel f) or ten (panel g) pairings of a pre- (${t}_{{\rm{pre}}}$) and a postsynaptic (${t}_{{\rm{post}}}$) burst event at a temporal offset of $\varDelta T={t}_{{\rm{post}}}-{t}_{{\rm{pre}}}$. Each burst event has a duration of 1 s and contains ten smaller 50 ms-long events. The postsynaptic burst contributes an additive term $I(t)$ with amplitude ${B}_{{\rm{amp}}}$ to the postsynaptic calcium, ${\tau }_{Y}\frac{d{Y}_{k}}{{dt}}=-{Y}_{k}(t)+{B}_{{\rm{amp}}}I(t)$. Note that in comparison to the calcium released by stimulating a synapse (Eq. 4), here ${B}_{{\rm{amp}}}$ provides a stronger contribution to the postsynaptic calcium (Table 1). We assumed that this postsynaptic signal comes from a bAP and hence is stronger than the synaptic signal^48^. We indirectly varied ${B}_{{\rm{amp}}}$ in Supplementary Fig. 9 where we simulated Eq. (19) on a dendritic tree. We found that significantly weakening the calcium signal from direct postsynaptic stimulation preserves the shape of the BTDP curve (including the timing requirements for potentiation and depression) but makes it somewhat flatter.

For the simulations in Fig. 2b, c, we distributed $N=\lfloor L\nu \rfloor$ synapses on a branch of length $L$, where $\lfloor x\rfloor$ is the largest integer smaller or equal to $x$ and where we choose $L$ sufficiently long ($30$ ${\rm{\mu }}{\rm{m}}$ in b and 64 ${\rm{\mu }}{\rm{m}}$ in c) to avoid confounds from periodic boundary conditions. We varied the density $\nu$ between 0.05 and 0.75 μm^−1^ to capture random and systematic fluctuations in local synaptic density^98^. We generated 12 min in Fig. 2b (4 h in Fig. 2c) of correlated Poisson event trains^99^, homogeneous across pairs, and with a fixed firing rate of 15 min^−1^. To compute the instantaneous change in synaptic efficacy in Fig. 2b, we fixed the synaptic efficacy to the initial value and used Eq. (10) to compute the change from the same initial efficacy averaged over 12 min, as a proxy for the ensemble average over inputs. In Fig. 2c, the synaptic efficacy was not fixed to the initial value and evolved according to Eq. (10). For the simulations in Fig. 2d, we used an initial efficacy of 0.9 and, in line with the experimental paradigm^6^, generated 6 min of correlated Poisson input for synapses distributed at a high density ($\nu =0.5$ ${\rm{\mu }}{{\rm{m}}}^{-1}$) or low density ($\nu =0.05$ ${\rm{\mu }}{{\rm{m}}}^{-1}$).

In Figs. 3 and 4, we simulated a branch with length *L* of over 15 days. All synaptic receptive fields were initialized with an orientation drawn from a uniform distribution over the interval $[{0}^{\circ },{360}^{\circ }]$. For the simulations in Figs. 5 and 6, we tested a morphologically realistic dendrite model by using a reconstructed pyramidal cell from layer 2/3 of the mouse visual cortex (Allen Cell Type database, ID 502269786) which we resampled into equally sized segments of $10$ ${\rm{\mu }}{\rm{m}}$ using the TREES toolbox^100^. We used this dendritic tree for both types of simulations, ferret and mouse, since to our knowledge no morphological reconstruction of a pyramidal layer 2/3 neuron from the ferret visual cortex is openly available. We do not expect this to influence our results, since the specific branching structure of the dendritic tree does not matter in our model. Our results depend on the anatomical argument of receptive field diameter and center spread and do not consider other factors, for instance, differences in morphology and electrophysiology across species. Furthermore, we did not include any differences between synaptic inputs on the apical vs. basal parts of the dendritic tree. Although apical and basal dendrites likely receive different inputs^101,102^, no location-dependent differences in orientation or overlap clustering have been reported^8,9^. Therefore, our model only considers synaptic differences pertaining to distance from the soma and their effect on global synaptic organization in the ferret and mouse. Because of the increased computational complexity, the duration for the simulations with the morphologically realistic dendrite model was 5 days, which is sufficient for most of the synapses to reach a stable state.

For the simulations with spike-timing-dependent plasticity in Supplementary Fig. 5, we used faster time scales of our pre- and postsynaptic accumulators (${\tau }_{M}$ = 60 ms, ${\tau }_{Y}$ = 1 ms), and further changed $\eta$ = 0.3 and $\phi =\frac{3}{5}$ ms^−1^. As we demonstrated analytically in Supplementary Note 5, the issue with generating clustering using this rule is its short integration time window (tens of milliseconds) rather than the exact choice of parameters^33^.

### Statistics

All correlations were computed as Pearson correlation coefficients, i.e., for two random variables $X$ and $Y$ we compute $\frac{\langle (X-{\mu }_{X})(Y-{\mu }_{Y})\rangle }{{\sigma }_{X}{\sigma }_{Y}}$, where ${\rm{\mu }}$ and $\sigma$ denote the mean and the standard deviation. In Fig. 2b, for each pair of correlation and synaptic density we computed the average change in synaptic efficacy over all synapses in 50 simulations.

To compute the correlations in Figs. 3b, g and 4f, we first applied a boxcar filter of length 3 s to generate signals consistent with the slow calcium dynamics in experimental imaging studies^19^. The experiments from ref. ^6^ reproduced in Figs. 2e and 4f report coactivity—the fraction of events at a given synapse that occurs in concert with events at nearby synapses. Coactivity is closely related to the Pearson correlation coefficient when it is only applied to pairs of synapses (see Supplementary Note 6). To estimate the spatial decay of correlations as a function of distance, $\lambda$, in Supplementary Fig. 4, we computed the average correlation between pairs of synapses $\varDelta d$ apart, then subtracted the average correlation between pairs of synapses more than $50$ ${\rm{\mu }}{\rm{m}}$ apart and fit a Gaussian function with shape ${A}_{0}{\rm{exp }}(-{\varDelta d}^{2}/(2{\lambda }^{2}))$ to the resulting curve^9^.

We computed the receptive field overlap in Fig. 4e as the spatial receptive field correlation^8^, i.e., the pixel-wise Pearson correlation coefficient between Gabor filters associated with pairs of synapses. The correlation for different receptive field center spreads in Fig. 4h, i was computed by first multiplying the coordinates of the receptive field center position with a scalar and then calculating the overlap ${o}_{{{{ij}}}}$ between all pairs of synapses ${{i}}$ and ${{j}}$. Next we computed a weighted average overlap for each synapse with its neighbors using the proximity values ${s}_{{{{ij}}}}$ as $\mathop{\sum}_{j}{s}_{{{{ij}}}}{o}_{{{{ij}}}}/\mathop{\sum}_{j}{s}_{{{{ij}}}}$ and related it to the correlation, which is a monotonically increasing function of the overlap (Supplementary Fig. 7). In Supplementary Fig. 7, we generated a Gaussian noise image by drawing independent samples from a standard normal distribution and reshaping them into a matrix of the same shape as the receptive field filters (73 × 73 pixels). Then we applied a Gabor filter with random orientation, wavelength of 2 pixels per cycle and a ratio of the semi-major and semi-minor axes equal to $\frac{1}{2}$ to the resulting noise image. When computing the spatial overlap between receptive fields (Supplementary Fig. 7), we generated a filtered noise image for each receptive field and added the noise to the receptive field, scaled by a factor uniformly distributed between $\frac{1}{2}$ and 2.

The term orientation difference^8^ denotes the absolute difference in orientation between receptive fields of pairs of synapses modulo 180°,

19 $$\begin{array}{c}{\min }({\rm{|}}{\theta }_{i}^{{\prime} }-{\theta }_{j}^{{\prime} }{\rm{|}},{180}^{\circ }-{\rm{|}}{\theta }_{i}^{{\prime} }-{\theta }_{j}^{{\prime} }{\rm{|}}),\end{array}$$

where ${\theta }_{i}^{{\prime} }$ is defined as ${\rm{mod}}({\theta }_{i},{180}^{\circ })$. The term circular dispersion denotes the orientation difference between the receptive field of a given synapse and the soma, where the orientation preference of the soma is the circular average of all synaptic preferences^7^, ${\rm{arg }}\,\big(\frac{1}{N}{\sum }_{j=1}^{N}{e}^{i{\theta }_{j}}\big)$. We define the mean circular dispersion in Fig. 5i as the average circular dispersion of all synapses less than 50 μm apart. Analogously, the term receptive field offset denotes the Euclidean distance between the center of a given synaptic receptive field and the somatic receptive field center, defined as the average location of all synaptic receptive fields. The average circular dispersion and average receptive field offset in Figs. 5 and 6 were computed as the average over synapses within dendritic segments. The coaxial space^8^ (Fig. 6f, g) is defined as the portion of space up to 45° on either side of the axis extending along the average orientation of all synaptic receptive fields. Conversely, the orthogonal space is the remaining visual space that is not coaxial.

### Reporting summary

Further information on research design is available in the Nature Research Reporting Summary linked to this article.

## Supplementary information

Supplementary Information

Reporting Summary

## Data Availability

The morphological reconstruction that supports the findings of this study is available from the Allen Cell Type database, ID 502269786.

## Code availability

We used the openly available TREEs toolbox (https://www.treestoolbox.org, v1.15) and the toolbox for generating correlated Poisson trains (https://de.mathworks.com/matlabcentral/fileexchange/20591-sampling-from-multivariate-correlated-binary-and-poisson-random-variables, v1.0). To generate retinal waves, we used open source code (https://swindale.ecc.ubc.ca/home-page/software/retinal-wave-models/). We extracted all displayed experimental data from the appropriately referenced publications using open source software^103^. The code for this modeling study was written by Jan H. Kirchner using the MATLAB 2019a programming environment. The relevant code to generate the figures of this paper is publicly available at https://github.com/comp-neural-circuits/synaptic-organization (10.5281/zenodo.4630142).
