# Supplementary material for: Emergence of local and global synaptic organization on cortical dendrites
Source: Nat Commun. 2021 Jun 28;12:4005. doi: 10.1038/s41467-021-23557-3 (PMC8239006; doi:10.1038/s41467-021-23557-3)
Supplement: Supplementary file 1 — Supplementary Information [file 41467_2021_23557_MOESM1_ESM.pdf]

Supplementary Information

Emergence of local and global synaptic organization on cortical dendrites

Jan H. Kirchner[1,2] & Julijana Gjorgjieva[1,2,*]

[1]*Computation in Neural Circuits Group, Max Planck Institute for Brain Research, 60438 Frankfurt, Germany*

[2]*School of Life Sciences, Technical University of Munich, 85354 Freising, Germany;*

**Corresponding author. E-Mail: gjorgjieva@brain.mpg.de*

Supplementary Figures

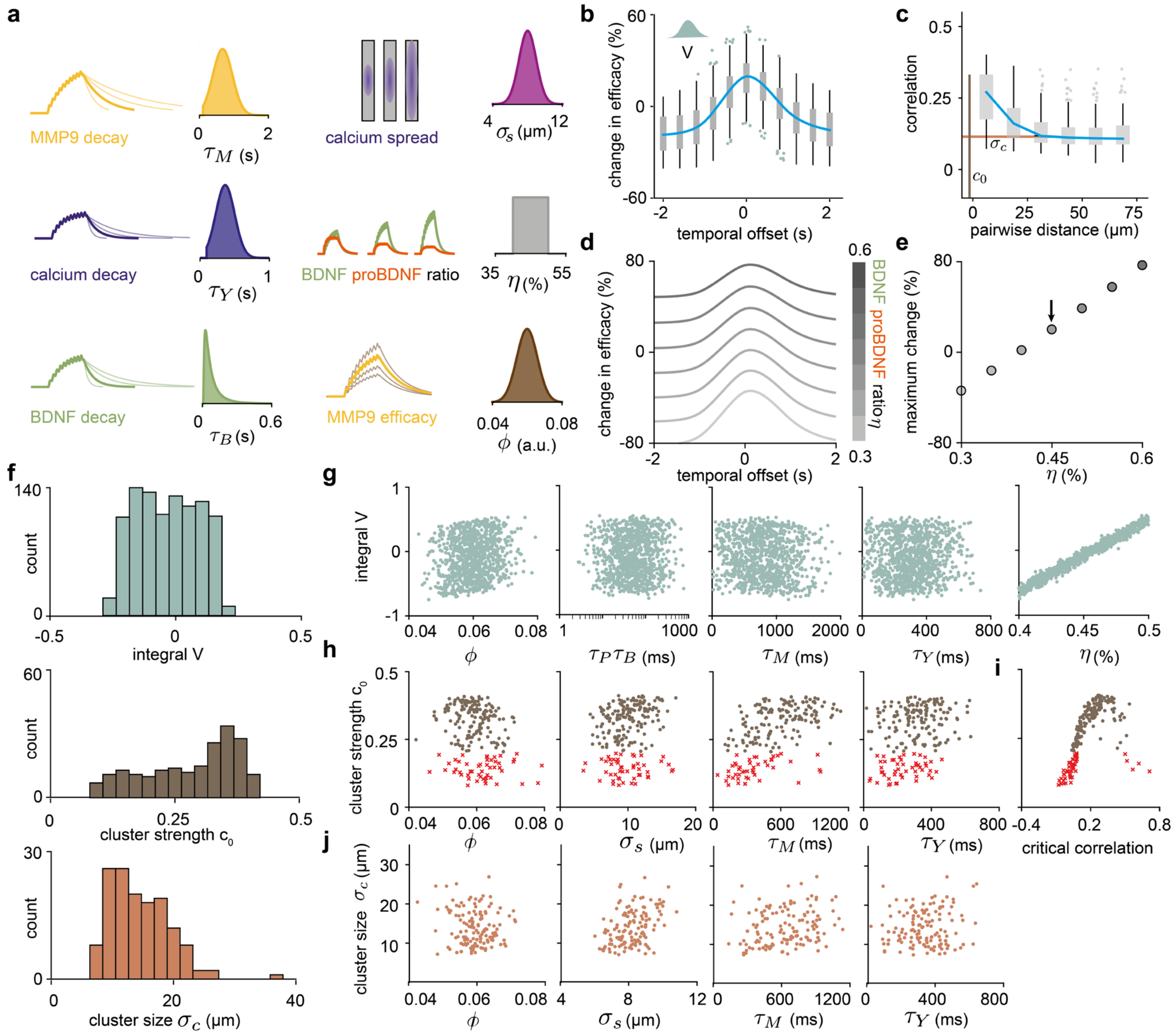

Supplementary Figure 1: **Uncertainty quantification and sensitivity analysis of the shape of the BTDP rule and the emergence of synaptic clustering.** (**a**) Illustration of prior distributions over the model parameters (see Table 1 and Methods) in the sensitivity analysis. The parameter is denoted below the distribution. Schematics on the left show how each parameter affects the accumulators. (**b**) Boxplot of changes in synaptic efficacy for different temporal offsets between pre- and postsynaptic bursts (n = 1,000 simulations with parameter distributions as in **a**). Blue line indicates the median, the box is drawn between the 25th and 75th percentile, whiskers extend

above and below the box to the most extreme data points that are within a distance to the box equal to 1.5 times the interquartile range and points indicate data points outside that range. Inset: the integral over the learning window, V (used in **f**,**g**). (**c**) Correlations for pairs of synaptic inputs in the model driven by retinal waves as a function of synaptic distance (same simulations as **b**). Horizontal brown line illustrates the cluster size σ_c (used in **f**,**j**), while the vertical brown line illustrates the cluster strength c_0 (defined to be the average correlation at distance 0-5 μm and used in **f**,**h**,**i**). Boxplots are defined as in **b**. (**d**) Change in efficacy for different temporal offsets between pre- and postsynaptic bursts with color indicating different initial ratios of BDNF to proBDNF (η). (**e**) Maximum change in efficacy from **d** as a function of η. Arrow indicates the value of η used in this study. (**f**) Histograms of V (top, see **b** for definition), c_0 (middle, see **c** for definition) and σ_c (bottom, see **c** for definition) from all 1,000 simulations. (**g**) V (see **b** for definition) as a function of the parameter values drawn from the distributions in **a** except σ_s, which has no effect in the BTDP stimulation protocol. (**h**) Same as **g** for c_0 (see **c** for definition) but without the parameter η, since small variations in η can abolish clustering. Red crosses indicate simulations with poor clustering ($c_0 < 0.2$). (**i**) Cluster strength c_0 (see **c** for definition) as a function of the critical level of correlation, c^*. (**j**) Same as **h** for cluster size σ_c (see **c** for definition).

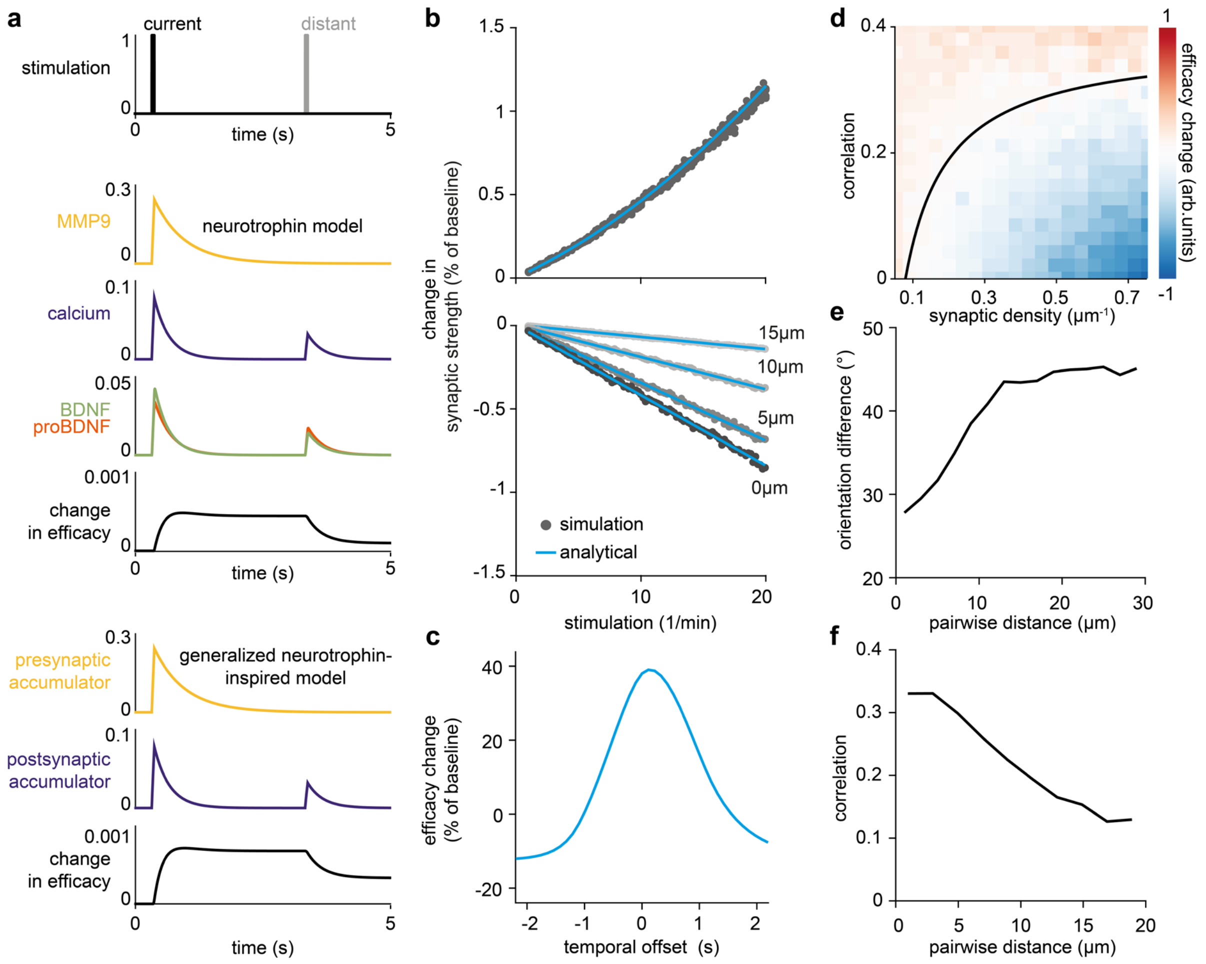

Supplementary Figure 2: **The generalized neurotrophin-inspired model captures the key properties of the full neurotrophin model.** (**a**) Traces of modeled variables (MMP9 and calcium for the neurotrophin model, and pre- and postsynaptic accumulator for the generalized model) associated with one synapse following the stimulation of that synapse (top row, current synapse) or of another synapse stimulated 3 seconds later (top row, distant synapse). The variables fill the same functional role generating a similar change in efficacy. (**b**) Percentage change in synaptic efficacy (of baseline) of the stimulated synapse (top) and the unstimulated synapse (bottom) as a function of input rate (in bursts per minute, after 1 min of continuous stimulation) and distance. As in Figure 1d but with simulations from the generalized neurotrophin-inspired model. (**c**) Percentage change in synaptic efficacy (of baseline) as a

function of temporal offset. As in Figure 1g but with simulations from the generalized neurotrophin-inspired model. (**d**) Average instantaneous change in synaptic efficacy as a function of input correlation and density. As in Figure 2b but for the neurotrophin model. (**e**) Orientation difference between pairs of synapses as a function of distance. As in Figure 3f but for the neurotrophin model. (**f**) Correlation between pairs of synaptic inputs as a function of distance. As in Figure 3g, but for the neurotrophin model.

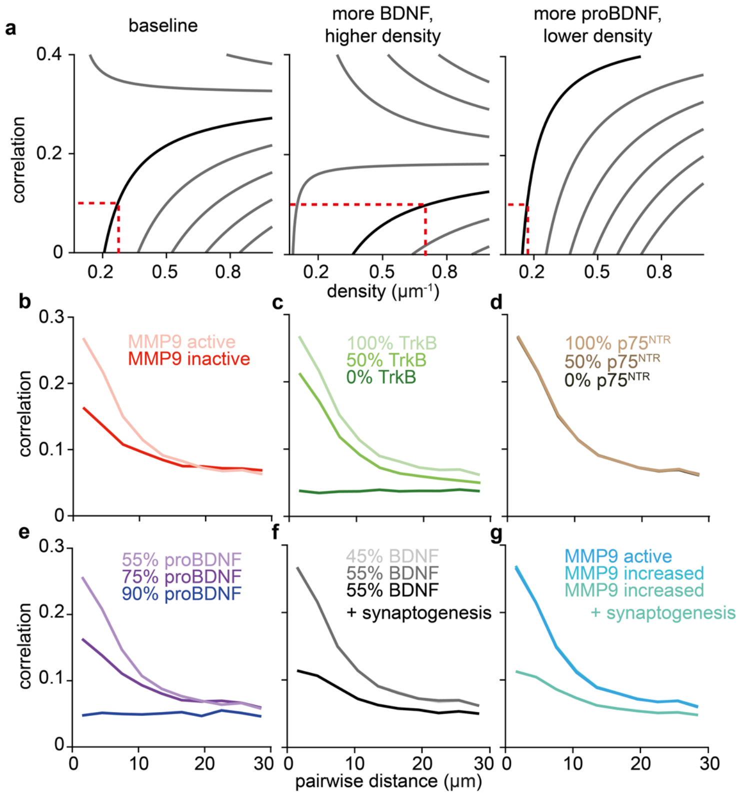

Supplementary Figure 3: **Established synaptic clustering can be rapidly diminished when different aspects of neurotrophin interactions are perturbed.** (**a**) Contour plots of change in synaptic efficacy as a function of synaptic density and input correlation as in Figure 2b for different ratios of BDNF to pro-BDNF. Red dashed line indicates densities at which change in synaptic efficacy is stabilized for a fixed level of correlation. (**b-g**) Correlation between pairs of synaptic inputs as a function of distance for initially clustered branches (lightest shade) that are subsequently stimulated for an additional hour with retinal wave input and with modified model parameters (darker shades). Perturbed parameters are: (**b**) decreasing the MMP9 efficacy ϕ, (**c**) the effectiveness of TrkB activation α, (**d**) effectiveness of $P75^{NTR}$ activation β, (**e**) the ratio of proBDNF to BDNF η in favor of proBDNF, (**f**) the ratio of proBDNF to BDNF η in favor of BDNF and with twice the density of active synaptic inputs[10], (**g**) increasing the MMP9 efficacy ϕ with a potentially increased density of active synaptic inputs.

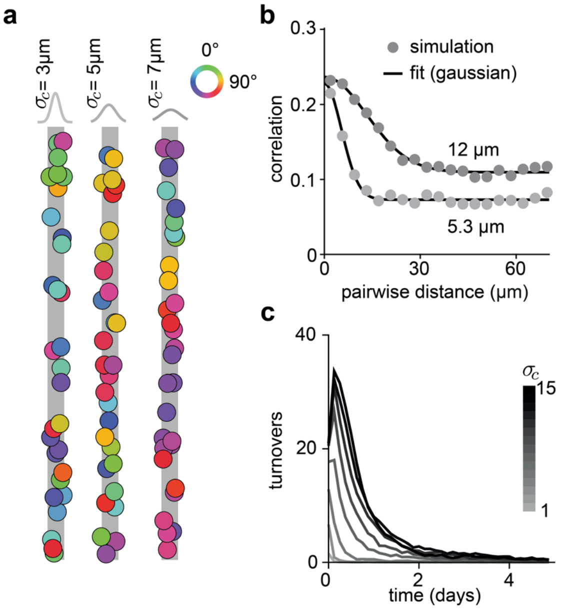

Supplementary Figure 4: **Postsynaptic calcium spread constant determines cluster size**. (**a**) Three examples of clustered dendritic branches from simulations with receptive field spread as in ferret with different postsynaptic calcium spread constants (top). (**b**) Correlation between pairs of synaptic inputs as a function of distance for two different values of the postsynaptic calcium spread constant ($\sigma_c = 5$ μm and $\sigma_c = 12$ μm). Solid lines are the best Gaussian fit. (**c**) Number of turnovers per day of simulation for different postsynaptic calcium spread constants.

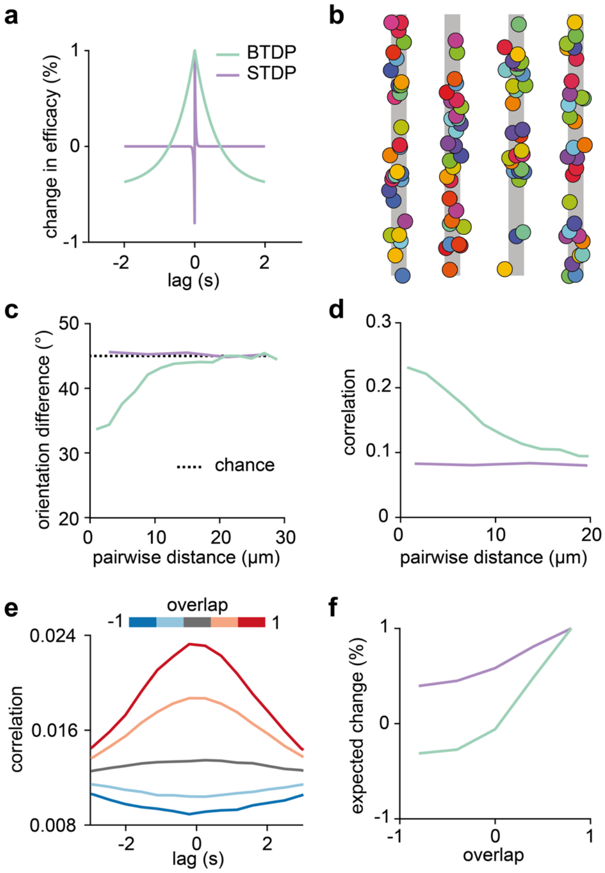

Supplementary Figure 5: **Spike-timing-dependent-plasticity (STDP) does not generate synaptic clustering under retinal wave input due to the mismatch of timescales**. (**a**) Change of synaptic efficacy (also called the learning window) as a function of the time lag between pre- and postsynaptic bursts for BTDP and spikes for STDP. Note the big difference in timescales. (**b**) Four examples of branches after plasticity based on STDP that do not show orientation clustering. (**c,d**) Orientation difference (**c**) and correlation (**d**) between pairs of synaptic inputs as a function of distance in the model with STDP (purple) and BTDP (green). (**e**) Cross-correlation at different time lags between pairs of spike trains from receptive fields with positive (red), no (grey) or negative (blue) overlap. (**f**) Expected change in synaptic efficacy, computed as the integral over the product of the cross-correlation function and the learning windows from **a** for different values of overlap in **e**. The quantity is normalized to the maximum change achieved for completely

overlapping receptive fields (overlap of 1). Note that the STDP rule yields the uniform potentiation or depression of weights, independent of overlap, while the BTDP rule generates synaptic competition depending on the overlap of receptive fields, see also Supplementary Note 5.

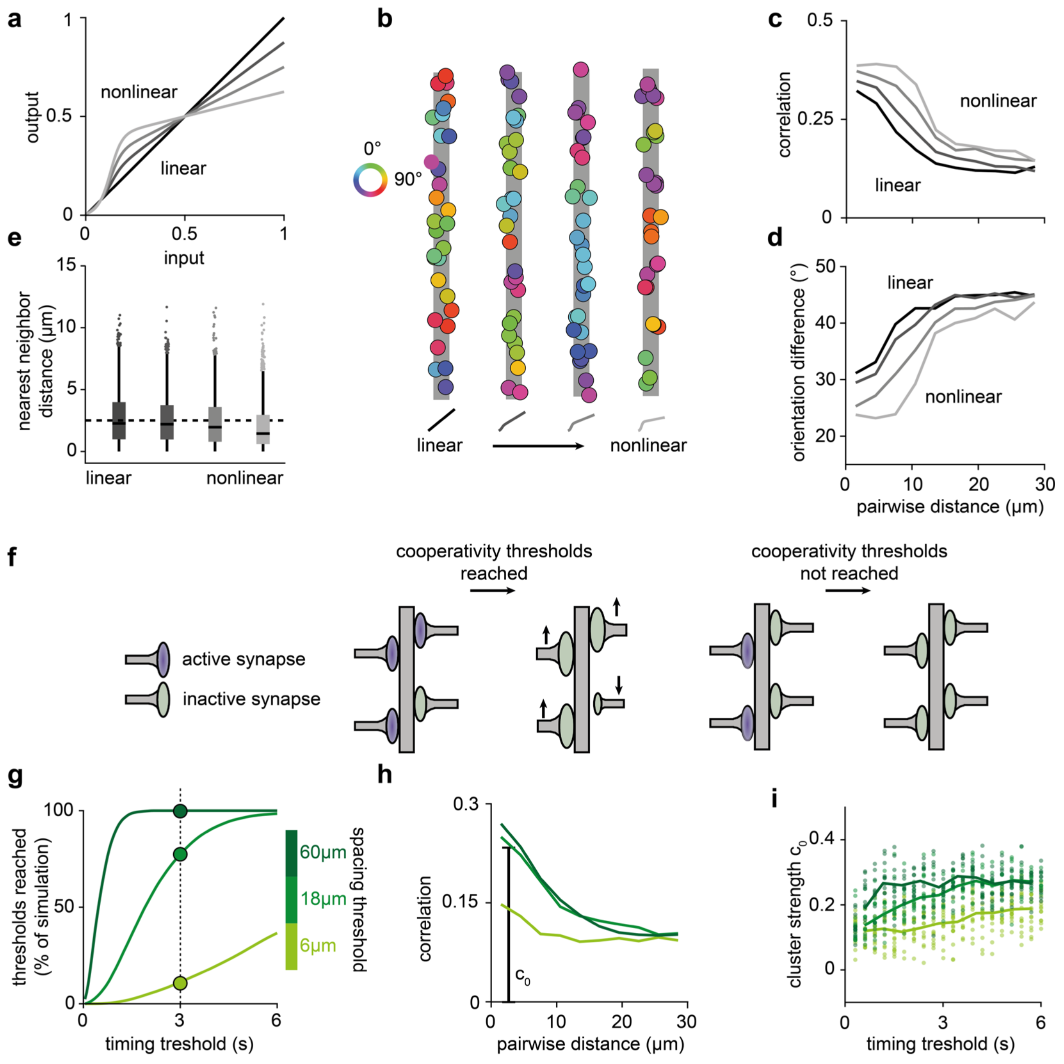

Supplementary Figure 6: **The effect of dendritic nonlinearities and cooperative plasticity on the emergence of synaptic clustering.** (**a**) Four dendritic nonlinear activation functions from linear (black) to nearly sigmoidal (light grey; compare for e.g. ref. [67]). (**b**) Four examples of orientation clustering of synaptic inputs after 15 days of retinal wave stimulation while varying the amount of nonlinearity. (**c,d**) Correlation (**c**) and difference in orientation preference (**d**) between pairs of synaptic inputs as a function of distance. (**e**) Distributions of nearest neighbor distance while varying the amount of nonlinearity. Horizontal line indicates the median, the box is drawn between the 25th and 75th percentile, whiskers extend above and below the box to the most extreme data points that are within a distance to the box equal to 1.5 times the interquartile

range and points indicate data points outside that range. Dashed line indicates mean distance for randomly distributed synapses. n = 10,800 pairs from 360 simulations. (**f**) Schematic illustrating that under cooperative plasticity synaptic efficacy changes only when at least three synapses within a distance smaller than a given spacing threshold become activated within a given timing threshold. (**g**) Percentage of simulation averaged over all synapses during which the spacing and timing cooperativity thresholds are reached as a function of the timing threshold. Different colors indicate different spacing thresholds. (**h**) Correlation as a function of synaptic distance for different spacing thresholds as in **g**. Timing threshold is fixed at 3 s. (**i**) Cluster strength c_0 (see **h**, for spacing threshold 18 μm) as a function of the timing threshold for different spacing thresholds (1,693 simulations). Different colors indicate different spacing thresholds.

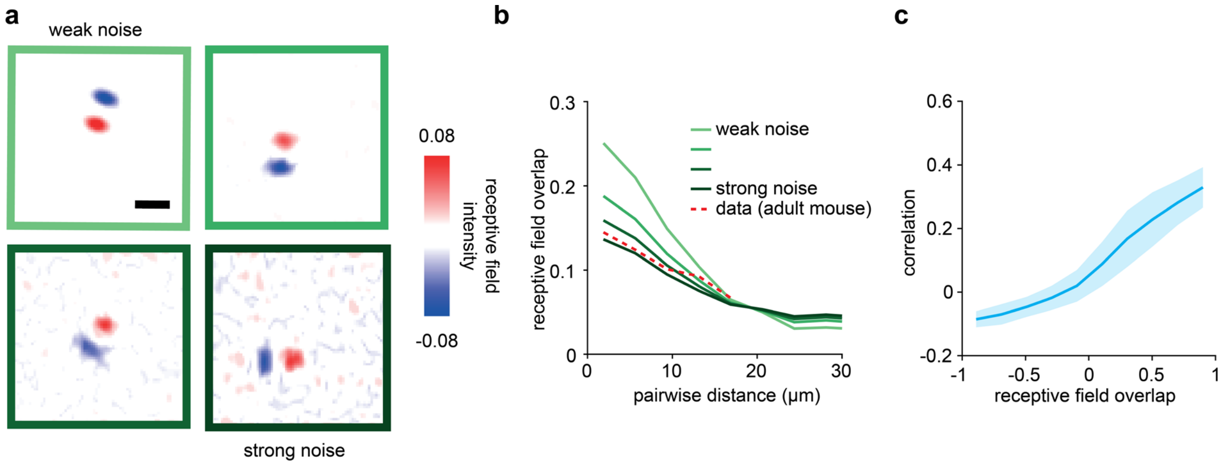

Supplementary Figure 7: **Adding noise to the Gabor receptive fields attenuates receptive field overlap.** (**a**) Four examples of simulated Gabor receptive fields with receptive field spread as in mouse[8] with different intensity of added noise (different shades of green). Scale bar is 20° in visual space. (**b**) Receptive field overlap for pairs of synaptic inputs as a function of distance. As Figure 4e but with receptive field overlap computed after adding noise of varying intensity. (**c**) Mean correlation between pairs of synaptic inputs as a function of receptive field overlap. Shaded region indicates one standard deviation.

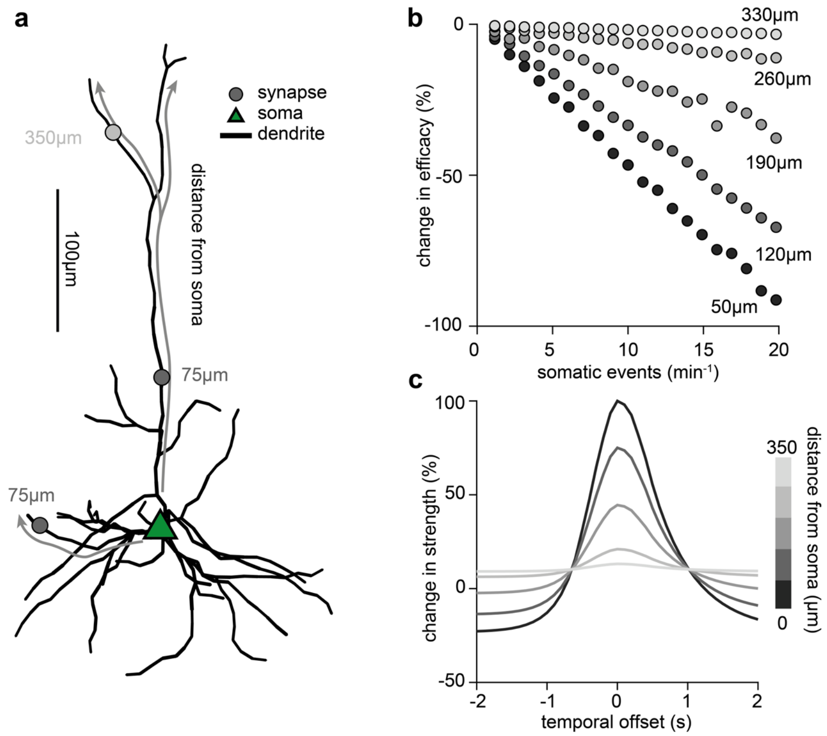

Supplementary Figure 8: **Including backpropagating action potential (bAP) preserves distance-dependent competition and the BTDP rule in the neurotrophin model.** (**a**) Synapses on the dendritic tree at different distances from the soma as in Figure 5 and Figure 6 under bAP stimulation at different rates. (**b**) Change in synaptic efficacy at an unstimulated synapse as a function of input rate (in somatic events per minute) and for different distances along the dendrite (note the extended scale on the colorbar compared to Figure 1d, bottom). (**c**) Percentage change in synaptic efficacy as a function of temporal offset for synapses at different distances along the dendrite. Plasticity is attenuated for synapses far away from the soma.

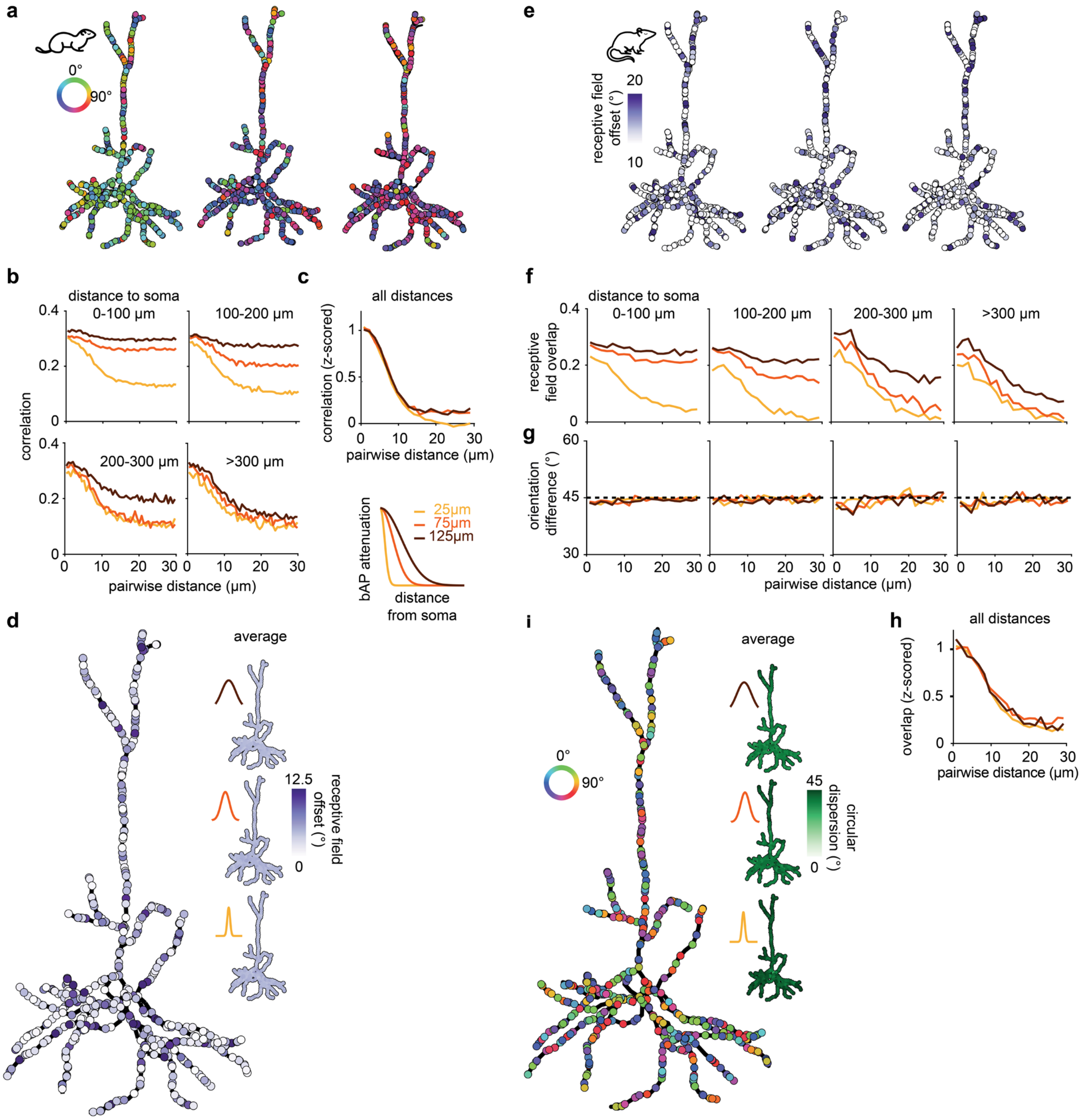

Supplementary Figure 9: **Correlations between pairs of synapses vs. distance in the presence of bAPs in a morphologically reconstructed L2/3 neuron.** Panels are generated from simulations with the receptive field spread and diameter as in ferret (**a-d**) or in mouse (**e-i**). (**a**) Three examples of clustered dendritic trees with a bAP attenuation factor of 75 μm. (**b**) Correlations between pairs of synaptic inputs as a function of distance at different positions along the tree (columns) and for different bAP attenuation factors. (**c**) Correlations with a normalized

baseline and peak correlation per simulation as a function of distance, pooled across all simulations and distances from the soma. (**d**) No emergence of global organization of receptive field center offset on the reconstructed pyramidal cell with synapses with a small receptive field center spread corresponding to ferret. Color indicates the receptive field center offset of the associated receptive field. Inset shows the receptive field center offset averaged over 62 simulations for the three different attenuation factors from Figure 5 (25 μm, 75 μm,125 μm). (**e**) As **a** but for receptive field offset. (**f,g**) As **b** but for receptive field overlap (**f**) and difference in orientation preference (**g**). (**h**) As (**c**) but for receptive field overlap. (**i**) No emergence of orientation preference on the reconstructed pyramidal cell with synapses with a large receptive field center spread corresponding to mouse. Color indicates the orientation preference of the associated receptive field. Inset shows the circular dispersion averaged over 80 simulations for the three different bAP attenuation factors from Figure 5 (25 μm, 75 μm,125 μm).

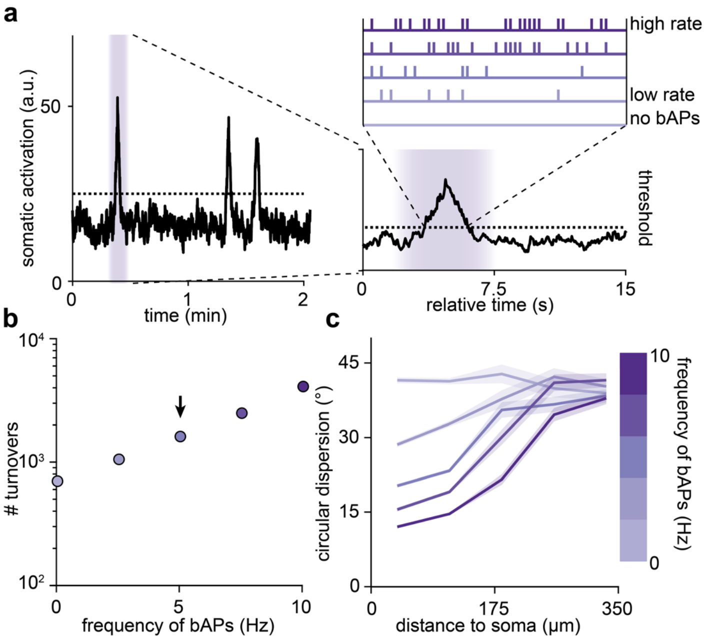

Supplementary Figure 10: **The number of backpropagating action potentials controls the degree of branch homogeneity.** (**a**) Schematic illustrating how the somatic accumulator (left) can generate either one or multiple bAP events (right) following sustained stimulation depending on the probability of generating a bAP in each time step. (**b**) Total number of turnovers after five days of retinal wave stimulation as a function of the frequency of bAPs per second when the somatic signal is above threshold. Arrow marks the frequency of bAPs used for Figure 5 and Figure 6. (**c**) Average circular dispersion as a function of the distance from the soma for different bAP frequencies. Shaded areas indicate 95% confidence intervals around the mean.

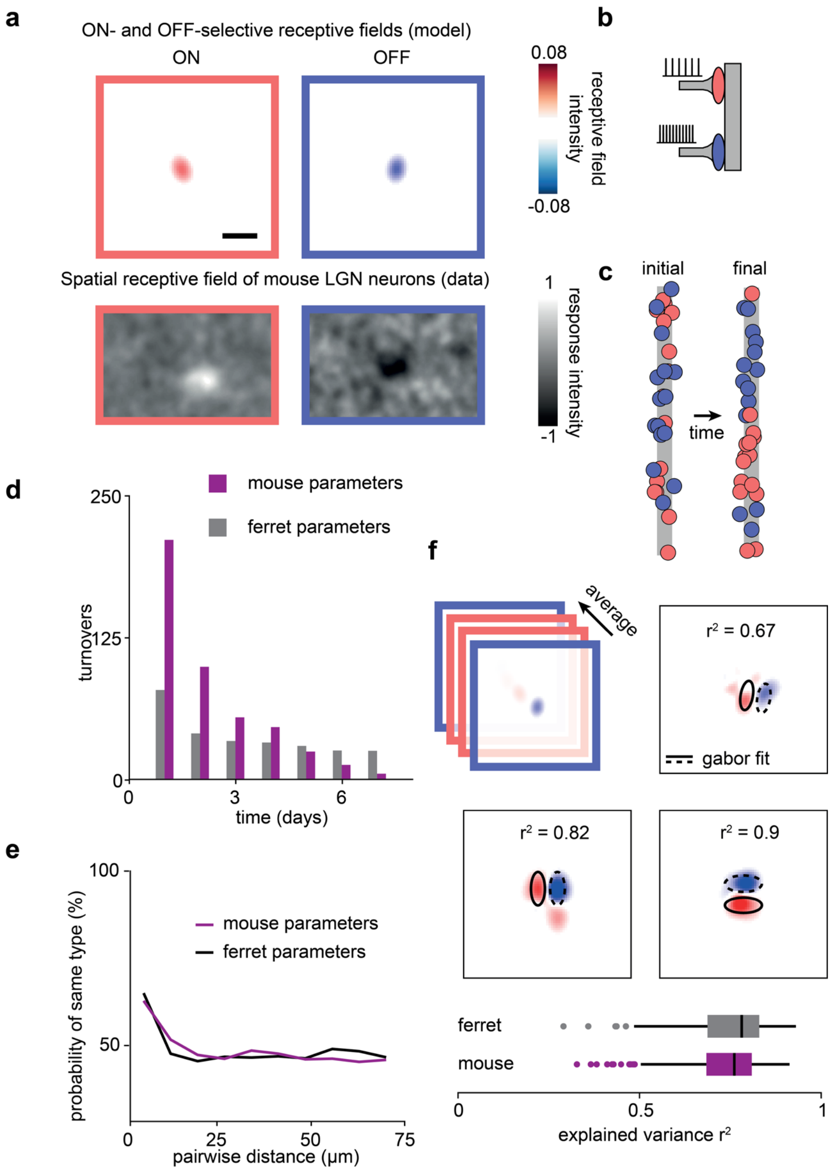

Supplementary Figure 11: **Generating Gabor receptive fields from ON- and OFF- selective thalamocortical inputs**. (**a**) Top: Examples of ON- and OFF- selective spatial receptive fields in our model. Scale bar is 20° in visual space. Bottom: Experimentally estimated receptive fields from adult mouse LGN from Tang et al. (*PLoS One,* 2016, ref. [87]) used under CC-BY (https://creativecommons.org/licenses/by/4.0/). (**b**) Following experimental data[88], we fixed the spontaneous firing rate of OFF-selective synapses to twice the rate of ON-selective synapses. (**c**) Example of ON-OFF clusters generated in one simulation. (**d**) Number of turnovers as a function

of time for simulations with receptive field center spread and diameter corresponding to mouse and ferret. (**e**) Probability of a pair of synapses being both ON- or both OFF-selective as a function of synaptic distance. (**f**) Top: Schematic illustrating how we compute the postsynaptic receptive field by averaging all synaptic receptive fields[104] and three examples of resulting Gabor receptive fields from different simulations. Bottom: Distributions of the variance that is explained by a Gabor fit, r^2, for simulations with large and small receptive field center spreads and diameters corresponding to mouse and ferret, respectively. Vertical line indicates the median, the box is drawn between the 25th and 75th percentile, whiskers extend left and right of the box to the most extreme data points that are within a distance to the box equal to 1.5 times the interquartile range and points indicate data points outside that range. n=300 simulations for each ferret and mouse.

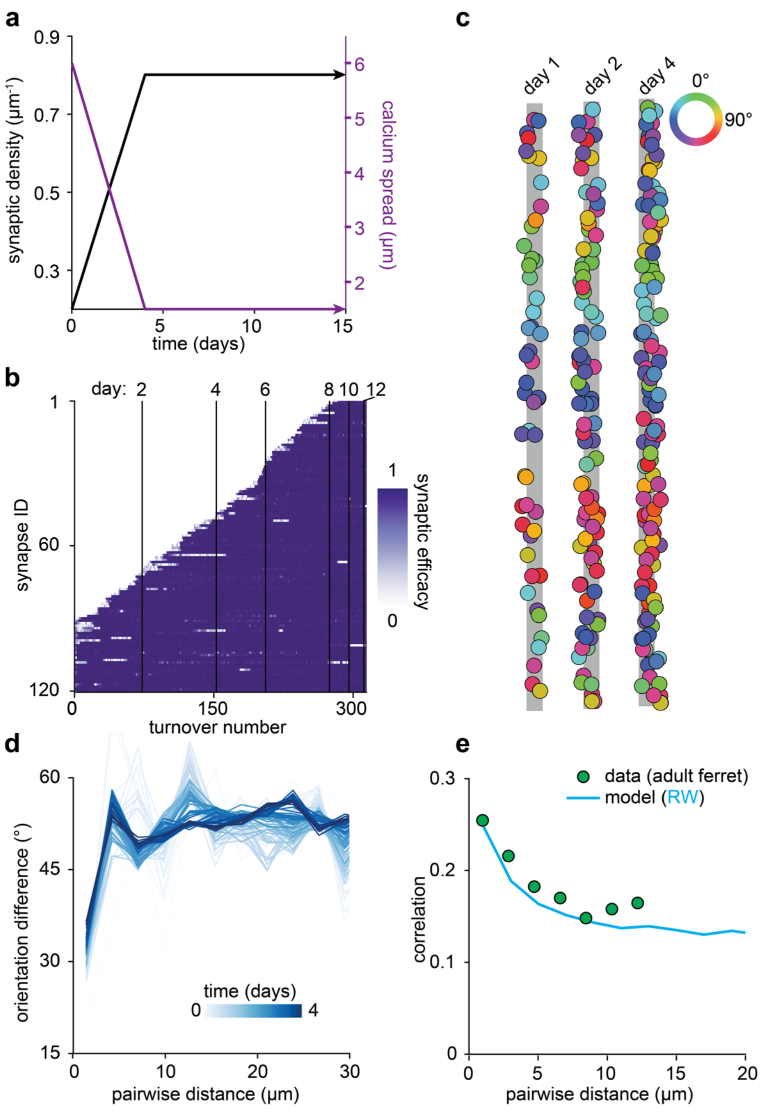

Supplementary Figure 12: **Synaptic clusters also form when synaptic density increases rapidly during development**. (**a**) Density of synapses per micron (left axis) and postsynaptic calcium spread constant (right axis) as a function of time from ref. [21]. (**b**) Synaptic efficacy of all synapses (rows) as a function of simulation time (columns). (**c**) Three examples of clustered branches at different time points in the simulation. (**d**) Orientation difference between pairs of synaptic inputs as a function of distance. Color indicates time in the simulation. (**e**) Correlation between pairs of synaptic inputs as a function of distance at the end of the simulation (data from adult ferret visual cortex reproduced from ref. [9]).

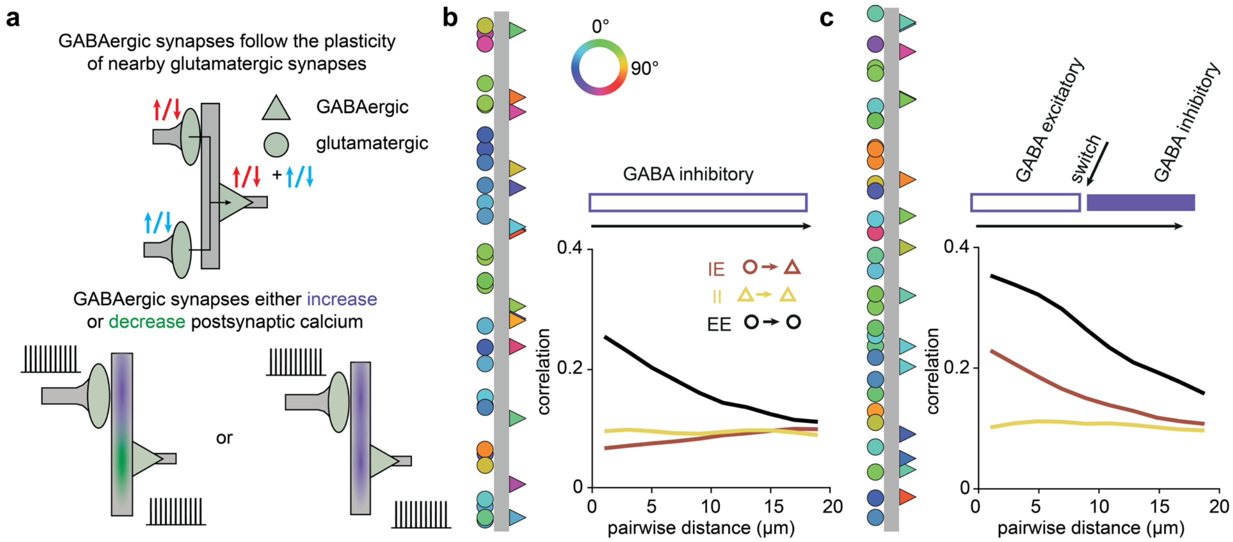

Supplementary Figure 13: **Inhibitory synapses provide a backbone for the formation of excitatory clusters.** (**a**) Plasticity of inhibitory (GABAergic) synapses is guided by the dynamics of surrounding excitatory (glutamatergic) synapses (top). Inhibitory synapses can decrease postsynaptic calcium if they are positioned sufficiently close to an active excitatory synapse (bottom). (**b**) Left: One example of a linear dendritic branch with GABAergic synapses that are inhibitory throughout the simulation. At the end of the simulation the branch exhibits clustered excitatory synapses tuned to the orthogonal orientation of nearby inhibitory synapses. Right: Correlations between pairs of synapses as a function of the distance. (**c**) As **b**, but with a developmental switch of GABAergic synapses from excitatory to inhibitory[73] (see Methods).

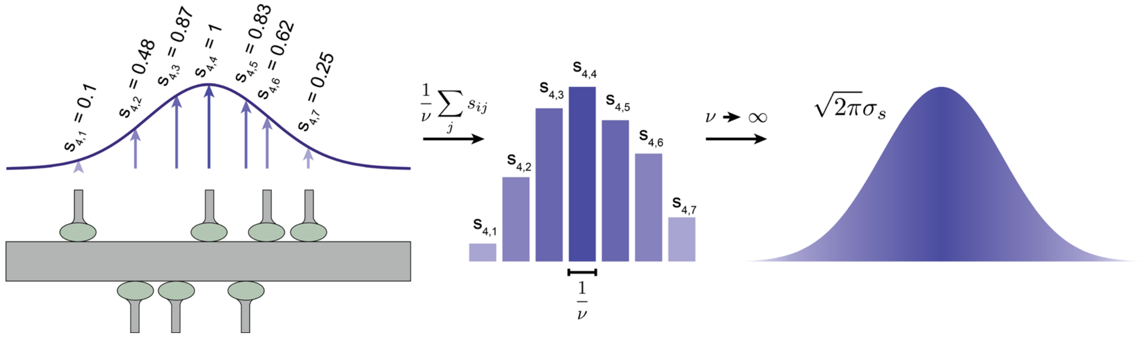

Supplementary Figure 14: **Approximating the value of the neighborhood interaction *S* with a Gaussian integral**. Left: One example of seven randomly distributed synapses alongside the Gaussian spread distribution (purple line) centered around synapse four and evaluated at all seven synapses (purple arrows). Middle: Sum over the individual contributions from all seven synapses multiplied with the average distance $\frac{1}{\nu}$. Right: Taking the limit $\nu \to \infty$ results in the integral over the initial spread distribution (purple volume).

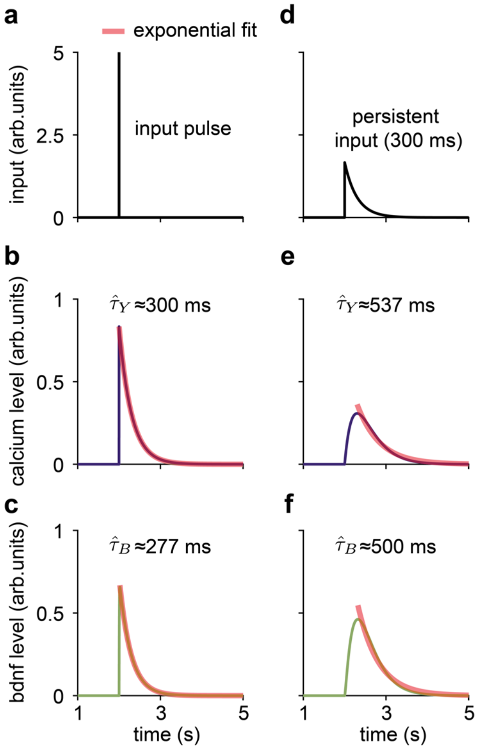

Supplementary Figure 15: **Fast neurotrophin time constants can generate effective slow decay time constants for different types of input.** An instantaneous input pulse (**a**) results in a rapid increase in postsynaptic calcium followed by a slow decay (**b**) which is mirrored by the extracellular BDNF level (**c**). In the case of temporally extended input (**d-f**), the decay of extracellular BDNF is even slower. In each case, the time constant of an exponential function fit to the decay of the accumulator is shown.

Supplementary Notes

1. Derivation of the generalized neurotrophin inspired model

To obtain a generalized version of the neurotrophin model we assume $P_k(t)$ and $B_k(t)$ to be in steady state $\frac{dP_k}{dt} = 0, \frac{dB_k}{dt} = 0$. This is justified since the corresponding time constants are very small compared to the time constants of M_k, Y_k or W_k. At steady state

$$\begin{aligned} P_k &= \frac{1-\eta}{1+M_k(t)} Y(t), \\ B_k &= \eta Y(t) + M_k(t) P_k = \eta Y(t) + M_k(t) \frac{1-\eta}{1+M_k(t)} Y(t). \end{aligned}$$

We can then rewrite the dynamics of the synaptic efficacy

$$\begin{aligned} \tau_W \frac{dW_k}{dt} &= \alpha\eta Y(t) + \alpha M_k(t) \frac{1-\eta}{1+M_k(t)} Y(t) - \beta \frac{1-\eta}{1+M_k(t)} Y(t) \\ &= Y(t) \left(\frac{\alpha M_k(t) - \beta}{M_k(t)+1} (1-\eta) + \alpha\eta \right). \end{aligned}$$

This has the form of a Hebbian learning rule, so we continue by making a first order Taylor approximation around $M_k = 0$, i.e. $\frac{\alpha M_k(t) - \beta}{M_k(t)+1} \approx (\alpha+\beta) M_k(t) - \beta$, and arrive at

$$\begin{aligned} \tau_W \frac{dW_k}{dt} &\approx Y(t)[((\alpha+\beta)M_k(t) - \beta)(1-\eta) + \alpha\eta] \\ &= Y(t)(\alpha+\beta)(1-\eta) \left(M_k(t) + \frac{(\alpha+\beta)\eta - \beta}{(\alpha+\beta)(1-\eta)} \right). \end{aligned}$$

Relabeling $v_k(t) = M_k(t)$, $u_k(t) = Y_k(t)$, $w_k(t) = W_k(t)$, $\rho = \frac{(\alpha+\beta)\eta-1}{(\alpha+\beta)(1-\eta)}$ and setting $\tau_w = \tau_W \frac{1}{(\alpha+\beta)(1-\eta)}$ allows us to write the generalized neurotrophin-inspired model as follows: a presynaptic accumulator

$$\tau_v \frac{dv_k}{dt} = -v_k(t) + \phi x_k(t),$$

a postsynaptic accumulator,

$$\tau_u \frac{du_k}{dt} = -u_k(t) + \sum_{l=1}^{N} s_{kl}\, w_l(t) x_l(t),$$

and a Hebbian equation for the change in synaptic efficacy,

$$\tau_w \frac{dw_k}{dt} = u_k(t)(v_k(t) + \rho).$$

2. Time and ensemble average analysis

To obtain an analytic expression for the expected change in synaptic efficacy, $\langle \dot{w}_k \rangle$, we insert Eq. 8 and Eq. 9 into Eq. 10 and assume separation of timescales where the synaptic efficacies change much slower than pre- and postsynaptic activity,

$$\tau_w \dot{w}_k = \phi \sum_l s_{kl}\, w_l \int_0^\infty \int_0^\infty x_l\,(t-r) x_k(t-s) \varepsilon_u(s) \varepsilon_v(r) dr\, ds$$
$$+\rho \sum_l w_l\, s_{kl} \int_0^\infty \varepsilon_u\,(s) x_l(t-s) ds,$$

and take the ensemble average and the average over time,

$$\lim_{T\to\infty}\frac{\tau_w}{T}\int_0^T\langle \dot{w}_k\rangle\,dt = \phi\sum_l s_{kl}\,w_l\int_0^\infty\int_0^\infty \underbrace{\lim_{T\to\infty}\frac{1}{T}\int_0^T\langle\, x_l(t-r)x_k(t-s)\rangle dt}_{\gamma_{kl}(r,s)}\ \varepsilon_u(s)\varepsilon_v(r)dr\,ds$$
$$+\rho\sum_l w_l\,s_{kl}\int_0^\infty \underbrace{\lim_{T\to\infty}\frac{1}{T}\int_0^T\langle\, x_l(t-s)\rangle dt}_{\mu_l}\,\varepsilon_u(s)ds,$$

where we obtain the firing rates μ_l and the raw cross-covariance $\gamma_{kl}(r,s)$ at lag τ so that we can write

$$\lim_{T\to\infty}\frac{\tau_w}{T}\int_0^T\langle \dot{w}_k\rangle\,dt = \phi\sum_l s_{kl}\,w_l\int_0^\infty\int_0^\infty \gamma_{kl}\,(r,s)\varepsilon_u(s)\varepsilon_v(r)dr\,ds + \rho\sum_l w_l\,s_{kl}\mu_l.$$

To simplify notation, the time integral $\lim_{T\to\infty}\frac{1}{T}\int_0^T\langle \dot{w}_k\rangle\,dt$ is absorbed into $\langle \dot{w}_k\rangle$. By making assumptions about the distribution of the input processes $x_k(t) = \sum_f \delta\,(t-t_k^f)$ we can further simplify the term $\int_0^\infty\int_0^\infty \gamma_{kl}\,(r,s)\varepsilon_u(s)\varepsilon_v(r)dr\,ds$. For the case of Poisson inputs with a Dirac delta cross-covariance function $\gamma_{kl}(r,s) = \mu_k\mu_l + \sqrt{\mu_k\mu_l}c_{kl}\delta(r-s)$, where c_{kl} is the zero-lag correlation, we can write

$$\int_0^\infty\int_0^\infty \gamma_{kl}\,(r,s)\varepsilon_u(s)\varepsilon_v(r)dr\,ds \quad = \mu_k\mu_l + \sqrt{\mu_k\mu_l}c_{kl}\underbrace{\int_0^\infty \varepsilon_u\,(r')\varepsilon_v(r')dr'}_{\frac{1}{\tau_u+\tau_v}}$$

so that

$$\tau_w\langle \dot{w}_k\rangle \quad = \phi\sum_l s_{kl}\,w_l\left(\mu_k\mu_l + \frac{\sqrt{\mu_k\mu_l}c_{kl}}{\tau_u+\tau_v}\right) + \rho\sum_l w_k\,s_{kl}\mu.$$

We also consider an example where non-zero-lag correlations are present. We construct the input process by smoothing a Poisson train of events with a boxcar filter with width x_{dur},

$$x_k(t) \quad = \int_0^\infty \sum_f \delta\,(s - s_k^f)(H(t-s) - H(t - x_{\text{dur}} - s))ds,$$

where $H(t) = \begin{cases} 0, & t < 0, \\ 1, & t \geq 0, \end{cases}$ is the Heaviside step function. Now the raw cross-covariance at lag t is given by

$$\gamma_{kl}(t) = \mu_k\mu_l + \sqrt{\mu_k\mu_l}c_{kl}x_{\text{dur}}\Theta(t),$$

where $\Theta(t)$ is the triangle function,

$$\Theta(t) = \begin{cases} 1 - \dfrac{|t|}{x_{\text{dur}}}, & |t| < x_{\text{dur}}, \\ 0, & \text{elsewhere.} \end{cases}$$

Consequently we can also compute $\int_0^\infty \int_0^\infty \gamma_{kl}\,(r,s)\varepsilon_u(s)\varepsilon_v(r)dr\;ds$ as

$$\begin{aligned} \int_0^\infty \int_0^\infty \gamma_{kl}\,(r,s)\varepsilon_u(s)\varepsilon_v(r)dr\;ds \quad &= \mu_k\mu_l + \sqrt{\mu_k\mu_l}c_{kl}x_{\text{dur}} \\ &+ \frac{\sqrt{\mu_k\mu_l}c_{kl}}{(\tau_u + \tau_v)}\left(\tau_u^2\left(e^{-\frac{x_{\text{dur}}}{\tau_u}} - 1\right) + \tau_v^2\left(e^{-\frac{x_{\text{dur}}}{\tau_v}} - 1\right)\right). \end{aligned} \tag{20}$$

3. Expected change in efficacy for the case of one stimulated and one unstimulated synapse

To obtain an analytic equation describing the amount of expected change in the simulations where only one synapse receives burst input (Figure 1d) of duration x_{dur}, we set $\mu_2 = 0$ and consequently $x_2(t) = 0$ and $\gamma_{22} = \gamma_{12} = \gamma_{21} = 0$ and simplify the expected change in synaptic efficacy of the inactive synapse as

$$\tau_w\langle\dot{w}_2\rangle = \underbrace{\phi\sum_l s_{2l}\,w_l\int_0^\infty\int_0^\infty\gamma_{2l}\,(r,s)\varepsilon_u(s)\varepsilon_v(r)dr\,ds}_{=0} + \rho\sum_l w_l\,s_{2l}x_{\mathrm{dur}}\mu_l$$
$$= \underbrace{x_{\mathrm{dur}}w_1\rho}_{K_3}\,\mu_1 \qquad \underbrace{s_{21}}_{exp(-{d_{12}}^2/(2\sigma_s^2))}.$$

Note, that here we used the fact that for a smoothed Poisson train x_1 with rate μ_1 and window size x_{dur} we can write $\langle x_1\rangle = \mu_1 x_{\mathrm{dur}}$. For the active synapse we use $c_{11} = 1$, $s_{11} = 1$ and simplify the expression for $\int_0^\infty\int_0^\infty\gamma_{kl}\,(r,s)\varepsilon_u(s)\varepsilon_v(r)dr\,ds$ computed in Eq. 20 for a Poisson train smoothed by a moving window of length x_{dur} to obtain

$$\begin{aligned}\tau_w\langle\dot{w}_1\rangle &= (\tau_u^2\left(e^{-\frac{x_{\mathrm{dur}}}{\tau_u}}-1\right)+\tau_v^2\left(e^{-\frac{x_{\mathrm{dur}}}{\tau_v}}-1\right))\frac{\phi w_1}{(\tau_u+\tau_v)}\mu_1 \\ &\quad +(x_{dur}\phi w_1 + K_3)\mu_1 + \phi w_1\mu_1^2 x_{\mathrm{dur}}^2 \\ &= K_1\mu_1 + K_2\mu_1^2,\end{aligned}$$

where K_1 and K_2 are newly defined constants. This equation provides the analytic solution in Supplementary Figure 2 and, up to a multiplicative scaling constant, Figure 1d.

4. Analytic condition for switch from depression-dominated to potentiation-dominated regime

To be able to analytically characterize the depression- and potentiation-dominated regimes (Figure 2b,c) we consider the completely homogeneous case with equal efficacies $w_k = w$ and rates $\mu_k = \mu$ for all k, and $c_{kl} = c$ for all pairs $k \neq l$. When using that $c_{kk} = 1$ and $s_{kk} = 1$, we can rewrite the expected change in efficacy as

$$\tau_w\langle\dot{w}_k\rangle = \phi w\left(\frac{c\mu}{\tau_u+\tau_v}+\mu^2\right)\underbrace{\sum_{k\neq l}s_{kl}}_{S_k-1} + \phi w\left(\frac{\mu}{\tau_u+\tau_v}+\mu^2\right) + \rho w\mu\underbrace{\sum_{k\neq l}s_{kl}}_{S_k-1} + \rho w\mu.$$

where we have defined $S_k = \sum_{l=1}^{N} s_{kl}$. Since $s_{kl} = e^{-\frac{d_{kl}^2}{2\sigma_s^2}}$, note that S_k is a sum over a Gaussian function centered at the position of synapse k, p_k, evaluated at the positions of all synapses, $p_1, \dots, p_N$. This observation led us to consider the limit where the number of synapses goes to infinity, $N \to \infty$, while the length of the dendrite stays fixed. In this limit, we can assume that all neighboring synapses are equidistant, $d_{k,k+1} = d_{l,l+1}$ for all pairs k, l, and interpret $\frac{L}{N} S_k = \frac{1}{\nu} \sum_{l=1}^{N} s_{kl}$ as a Riemann sum, where $\nu = \frac{N}{L}$ is the density of synapses. When taking the limit as N tends to infinity, ν also tends to infinity and we find that S_k can be approximated as the Gaussian integral (Supplementary Figure 14),

$$\lim_{N\to\infty} \frac{L}{N} \sum_{l=1}^{N} s_{kl} \quad = \int_{-\infty}^{\infty} e^{-\frac{-x}{2\sigma_s^2}} dx = \sqrt{2\pi}\sigma_s,$$

and therefore we can replace S_k by $\sqrt{2\pi}\sigma_s \nu$ and use the resulting expression to generate the contours in Figure 2b. Note that $\sqrt{2\pi}\sigma_s \nu$ depends on k through ν, which denotes the local density of synapses around synapse k; this is the same for all k in the homogenous case, therefore we let $S = S_k$ for all k, see Figure 2b. This allows us to write

$$\begin{aligned} \tau_w \langle \dot{w}_k \rangle \quad &\approx \phi w \left(\frac{c\mu}{\tau_u + \tau_v} + \mu^2 \right) (\sqrt{2\pi}\sigma_s \nu) + \phi w \left(\frac{\mu}{\tau_u + \tau_v} + \mu^2 \right) \\ &+ \rho w \mu (\sqrt{2\pi}\sigma_s \nu - 1) + \rho w \mu = K_4 c + K_5, \end{aligned}$$

where K_4 and K_5 are newly defined constants.

For the special steady-state case where the expected change in efficacy is zero, $\langle \dot{w}_k \rangle = 0$ for all k, we obtain the critical amount of pairwise correlation, c^*, at which the system switches between the potentiation- and the depression-dominated regime, $c^* = \frac{\kappa S - 1}{S - 1} = \frac{\kappa \sqrt{2\pi}\sigma_s \nu - 1}{\sqrt{2\pi}\sigma_s \nu - 1}$, with $\kappa = (-\frac{\rho}{\phi} -$

$\mu)(\tau_u + \tau_v)$. For high densities, $\nu \to \infty$, this fraction rapidly approaches κ, which thus represents an upper bound on the amount of correlation at which synapses experience uniform potentiation.

5. Analytic argument for why STDP cannot produce clustering in a developmental setting

Here we demonstrate that a plasticity rule like spike-timing-dependent plasticity rule (STDP), which operates on much faster time scales than the BTDP rule, cannot establish synaptic organization on dendrites when individual synaptic inputs are stimulated by bursts of action potentials from retinal waves. Assuming separation of timescales of neural activity and synaptic plasticity, a common assumption in analytical studies of synaptic plasticity[105], we can write the expected change in synaptic efficacy as a function of the learning rule $W(\Delta t)$ and the cross-correlation function $C(\Delta t, \Omega)$[33]

$$\langle \dot{w} \rangle \quad \propto \int_{-\infty}^{\infty} W\,(\Delta t) C(\Delta t, \Omega) d\Delta t,$$

where the STDP learning window is

$$W_{\mathrm{STDP}}(\Delta t) \quad = \begin{cases} A_{+} e^{-\frac{\Delta t}{\tau_{+}}}, & \Delta t \geq 0, \\ A_{-} e^{\frac{\Delta t}{\tau_{-}}}, & \Delta t < 0. \end{cases}$$

Note that here A_{+} and A_{-} denote the learning rates at zero offset $\Delta t = 0$, while τ_{+} and τ_{-} denote the timescales at which causal (pre-post) or acausal (post-pre) pairs affect potentiation vs. depression, respectively. The BTDP learning window can be described by the function

$$W_{\mathrm{BTDP}}(\Delta t) \quad = \begin{cases} (A_B + I) e^{-\frac{|\Delta t|}{\tau_B}} - I, & |\Delta t| \leq T_{\max}, \\ 0, & |\Delta t| > T_{\max} \end{cases}$$

where A_B denotes the amount of potentiation at zero offset $\Delta t = 0$, τ_B denotes the timescale at which pairs of pre- and postsynaptic activation produce potentiation, I denotes the maximum amount of depression for large Δt and $T_{\max}$ denotes the size of the learning window. Importantly, by definition the decay time constant of the BTDP learning window is much slower than that of the STDP learning window, $\tau_B >> \tau_+, \tau_-$ (a).

Due to the slow dynamics of retinal waves, the decay time constant of $C(\Delta t, \Omega)$ as a function of the time lag Δt is much slower than the decay time constant of the STDP learning window (a,e). Therefore, the cross-correlation is almost constant in the range where the STDP learning window is non-zero, $C(\Delta t, \Omega) \approx C(0, \Omega) > 0$, and the expected change in synaptic efficacy can be approximated by

$$\langle \dot{w} \rangle \quad \propto \underbrace{C(0, \Omega)}_{>0} (\tau_+ A_+ - \tau_- A_-).$$

Consequently, the change in synaptic efficacy is either uniformly positive or negative for all values of Ω and only depends on the parameters of the STDP learning window, $\tau_+ A_+ - \tau_- A_-$; this prevents selectivity where some efficacies potentiate and stabilize, while others depress.

In contrast, the BTDP rule is nonzero over an extended range and both cross-correlations at small and large lags can affect the change in efficacy. To investigate the effect of small and large lag contributions separately, we solve the equation $W_{\text{BTDP}}(t^*) = 0$ to obtain the critical lag at which the BTDP rule switches from potentiation to depression, $t^* = -\tau_B \log\left(\frac{I}{A_B + I}\right)$. Using the symmetry of both $C(\Delta t, \Omega)$ and $W_{\text{BTDP}}(\Delta t)$ in Δt we can then split the integral into a positive and a negative component,

$$\langle \dot{w} \rangle \quad \propto \underbrace{\int_0^{t^*} C\,(\Delta t, \Omega) W_{\mathrm{BTDP}}(\Delta t) d\Delta t}_{>0} + \underbrace{\int_{t^*}^{\infty} C\,(\Delta t, \Omega) W_{\mathrm{BTDP}}(\Delta t) d\Delta t}_{<0} \tag{21}$$

Importantly, due to the temporal dynamics of retinal waves, the dependence of the cross-correlation on the overlap Ω becomes smaller for large Δt. As a consequence, while the positive term of Eq. 21 depends strongly on Ω, the negative term depends much less on Ω. Thus, when the BTDP is parametrized appropriately, it selectively depresses synaptic inputs with poorly overlapping receptive fields (since the poor overlap leads to a small positive term in Eq. 21) and allows for the emergence of synaptic clustering.

6. Connection between coactivity and correlation

Under certain assumptions, we can establish a link between the coactivity of pairs of synapses used in experiments[6] (reproduced in Figure 2e and Figure 4f) report coactivity and pairwise Pearson correlation coefficient. When $x_1(t)$ and $x_2(t)$ are two binarized trains of activity taking the values 1 and 0 to indicate the presence or the absence of an event, then the coactivity of x_1 with x_2, $\mathrm{coac}(x_1, x_2)$, is defined as the percentage of the fraction of events in train x_1 that co-occur with events of the train x_2. We can write this compactly as $\mathrm{coac}(x_1, x_2) = \frac{\int_0^T x_1(t) x_2(t) dt}{\int_0^T x_1(t) dt}$.

Note that in general this is not symmetric, $\mathrm{coac}(x_1, x_2) \neq \mathrm{coac}(x_2, x_1)$. In our simulations, however, the firing rates are approximately equal, $\int_0^T x_1\,(t) dt \approx \int_0^T x_2\,(t) dt$, and therefore the coactivity is symmetric. Furthermore, we can write

$$\mathrm{coac}(x_1, x_2) = \frac{\int_0^T x_1\,(t) x_2(t) dt}{\int_0^T x_1\,(t) dt} \approx \frac{\langle x_1 x_2 \rangle}{\langle x_1 \rangle} = \frac{\mathrm{cov}(x_1, x_2)}{\mu_1} + \frac{\mu_1 \mu_2}{\mu_1},$$

where μ is the expected value of x, and $\mathrm{cov}(x_1, x_2)$ is the covariance between x_1 and x_2, $\mathrm{cov}(x_1, x_2) = \frac{1}{T}\int_0^T (x_1(t) - \mu_1)(x_2(t) - \mu_2)dt$. If now x_1 and x_2 are also Poisson, i.e. their mean equals their variance, $\mu_1 = \sigma_1^2 = \sigma_1\sigma_2$, and if we assume the firing rates to be small, then we can further simplify this as, $\mathrm{coac}(x_1, x_2) = \mathrm{corr}(x_1, x_2) + \mu_2 \approx \mathrm{corr}(x_1, x_2)$.

References

104. Sedigh-Sarvestani, M. *et al.* Intracellular, in vivo, dynamics of thalamocortical synapses in visual cortex. *J. Neurosci.* **37**, 5250–5262 (2017).

105. Gerstner, W. & Kistler, W. M. *Spiking neuron models: Single neurons, populations, plasticity*. (Cambridge University Press, 2002).
